# Innovating Electrosynthesis From Metal Electrodeposition to Organic Chemistry: Deep Eutectic Solvents as Efficient and Sustainable Media

**DOI:** 10.1002/cssc.70799

**Published:** 2026-06-14

**Authors:** Cristiana Margarita, Stefano Nejrotti, Alejandro Leal‐Duaso, Marta Feroci, Alessandra Sanson, Claudia Barolo, Matteo Bonomo

**Affiliations:** ^1^ Dipartimento di Scienze di Base e Applicate per l’Ingegneria (SBAI) Sapienza University of Roma Roma Italy; ^2^ Istituto di Scienza, Tecnologia e Sostenibilità per lo Sviluppo di Materiali Ceramici (ISSMC‐CNR) Faenza Ravenna Italy; ^3^ URT CAMPUS Dipartimento di Scienze Chimiche e Tecnologie dei Materiali (DSCTM‐CNR) Torino Italy; ^4^ Dipartimento di Chimica NIS e Centro di Riferimento INSTM University of Torino Torino Italy; ^5^ Instituto de Síntesis Química y Catálisis Homogénea (ISQCH‐CSIC) Faculty of Science University of Zaragoza Zaragoza Spain

**Keywords:** deep eutectic solvents, metal electrodeposition, nanomaterials, organic electrosynthesis, sustainable chemistry

## Abstract

Electrosynthesis is an ideal tool for achieving effective processes across various domains of organic and inorganic chemistry, while ensuring environmental and economic sustainability. However, due to the requirement of a conductive reaction medium, supporting electrolytes are needed in organic solvents or water. Furthermore, the nature of the solvent itself can constitute an issue, as volatile organic compounds can pose operational safety risks, their electrochemical stability can be limited, and their production mainly relies on fossil sources. The use of aqueous media is instead limited due to reagents’ solubility and potential interferences due to the reactivity of water. A valuable and innovative alternative to conventional solvents is represented by hydrogen bond acceptor–donor mixtures known as deep eutectic solvents (DESs). These systems are characterized by a liquid phase that exhibits a certain degree of supramolecular organization, have conductive nature, and their physicochemical properties are easily tuneable through engineering of chemical composition. In this review, we highlight advances in the use of DESs in electrosynthesis, from electro‐organic transformations to the preparation of metal nanoparticles and inorganic nanomaterials highly useful as electrocatalysts, with a critical focus on how these systems impact both the sustainability aspects and synthetic outcomes of the reported procedures.

## Graphical abstract

This review analyzes deep eutectic solvents (DESs) as efficient media for both organic and inorganic electrosynthesis. The improvements in terms of synthetic opportunities and sustainability are discussed, by exploring the multiple roles of DESs as solvents, electrolytes, and promoters, enabling effective and selective electrosynthesis under mild and environmentally friendly conditions.

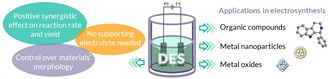

## 1 Introduction

Modern synthetic chemistry, across both its organic and inorganic domains, is compelled to meet criteria of environmental and economic sustainability, prompting the search for efficient and selective methods to achieve specific molecular architectures (for organic transformations) or materials’ features (for inorganic ones), while minimizing waste production and energy consumption [1, 2]. Electrochemical synthesis, or “electrosynthesis,” is an ideal tool in this regard, providing access to cleaner and milder processes [3, 4, 5]. First and foremost, in an electrochemically driven reaction, stoichiometric (or super‐stoichiometric) amounts of reducing or oxidising agents are replaced by electrons as clean reagents, improving atom economy, cost‐effectiveness, and safety by circumventing the need for hazardous chemicals. The concept is not strictly limited to redox reactions, as electric current can also be exploited to generate highly reactive intermediate species, thus acting in the capacity of a catalyst or promoter [6]. Notably, such electro‐generated intermediates are often obtained at relatively low temperatures and atmospheric pressure, thereby improving the energy efficiency and the feasibility of the synthesis, especially when the required electricity is derived from renewable sources [7]. Another highly relevant aspect in synthetic processes is the selectivity, which stems from the possibility of controlling key reaction parameters such as electrochemical potential or current density, which can be finely tuned for the desired oxidative or reductive process. In the synthesis of organic compounds, this is reflected in the minimization of unwanted side reactions [3, 4], lowering the waste associated with the procedure, or in other words, improving its mass efficiency [8, 9]. In inorganic synthesis, control over potential and current allows for directing the process towards specific compositions and morphologies, obtaining (in a reproducible way) materials with high purity and controlled size, such as nanoparticles (NPs) of uniform size distribution, or thin films of precise thickness [10].

Key components of an electrosynthesis working setup [11, 12], are the electrodes, at which the reaction takes place by transfer of electrons to or from the substrate, and the solvent—or rather, the solvent system. In fact, an electrochemically driven transformation requires that the conductivity of the reaction medium is high enough to ensure the current to flow at the operating conditions. Since commonly employed solvents (water or conventional organic solvents) do not meet this requirement, the actual solvent system is generally achieved by the dissolution of an inorganic or organic salt, inert towards the reagents and nonredox‐active at the operating potential, that acts as a supporting electrolyte [13, 14]. This implies the addition of materials that are not included into the synthesized products, thus significantly worsening the mass efficiency, the environmental impact, and the economic balance of the synthetic procedure. Although economically viable and ecologically friendly salts can be used, particularly for procedures carried out in water as the solvent (e.g., NaCl, KHCO_3_, and NaHCO_3_), they still represent a source of waste in the process. The recovery of the supporting electrolyte at the end of the reaction could mitigate its negative impact [13], but it is often not an easily viable option, especially when working with organic solvents and organic salts (typically, tetraalkylammonium tetrafluoroborates or hexafluorophosphates), due to the need to find a delicate balance between solubility of all components, separation of the product from the reaction mixture, and efficient recovery of the supporting electrolyte itself [15]. Although remarkable efforts have been made to develop supported [16, 17] or polymeric [18] electrolytes, this component still represents a source of waste and may impose additional purification steps to separate it from the product. In this context, transferring the synthesis from batch to flow configuration can eliminate, or strongly reduce, the need for a supporting electrolyte, since a short inter‐electrode distance is reflected in a drop in the ohmic resistance of the solution [19, 20, 21]. However, the use of flow chemistry might be limited by its technical demands, and, while highly valuable in delivering selective and efficient processes involving reactive intermediates and hazardous species in organic synthesis [22], it remains inapplicable, for instance, to the preparation of nanomaterials.

Apart from the need to employ an electrolytic additive, the nature of the solvent itself can constitute an issue in terms of sustainability. Indeed, electrochemical synthesis is often performed in volatile organic compounds (VOCs), such as acetonitrile, tetrahydrofuran, and *N*,*N*‐dimethylformamide, which pose operational safety risks, are harmful towards human health and the environment, and are generally produced from fossil sources [23, 24]. Furthermore, VOCs usually present strong stability limitations under the application of high electric currents, resulting in solvent degradation that burdens the energy efficiency and triggers purification issues. Water would be an ideal solvent, thanks to its abundance and inherent safety, and it has been investigated as a replacement for VOCs or in mixture with them [14], but its applicability is limited, beside its narrow electrochemical stability window, by the low solubility of many reagents, particularly in organic electrosynthesis, and by the potential interference with reaction pathways, arising from its own reactivity towards organic substrates and intermediates. Ionic liquids (ILs) have been extensively studied as alternative reaction media for electrosynthesis of both organic compounds [25, 26, 27] and inorganic materials [27, 28, 29, 30, 31] since their intrinsic ionic nature is suitable for circumventing the addition of the supporting electrolyte. ILs have been “historically” regarded as potentially sustainable solvents, thanks to their negligible vapour pressure and their thermal stability [32]. In addition to that, their physicochemical properties can be tailored by design of the molecular structure of their anion and cation [33, 34, 35]. However, it is nowadays widely recognized that ILs’ sustainability is heavily burdened by their high cost, resource‐consuming synthesis and purification, and toxicity towards human health and the environment [36, 37, 38].

**FIGURE 1:**
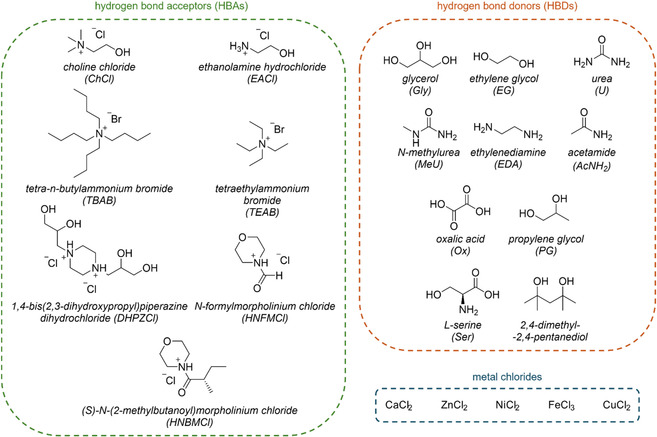
Compounds used to obtain the DESs presented in this review. Metal chlorides may interact with HBAs (as Lewis acids) or with HBDs (as HBAs).

In this context, a valuable alternative to conventional solvents and to ILs, for a plethora of applications, has been represented in the last two decades by the hydrogen bond acceptor–hydrogen bond donor (HBA:HBD) mixtures known as deep eutectic solvents (DESs) [39, 40, 41, 42]. These solvent systems are generally obtained by mixing two (or, in some cases, three) components in specific molar ratios, usually an ammonium salt and a polyol or an acid or an amine/amide (Figure 1), which interact mainly through hydrogen bonding to produce a liquid phase, usually characterized by a certain degree of supramolecular organization [43, 44, 45, 46, 47]. The scientific interest for DESs has been primarily fostered by their promising green features, specifically their low toxicity, negligible volatility, good biodegradability, feasible and 100% atom economic preparation, and potentially renewable sourcing [48]. These attractive characteristics are assumed from the fact that DES components do show, in most cases, a favourable toxicity/biodegradability profile, being plant metabolites [49, 50, 51] or renowned safe compounds, such as choline chloride (ChCl), the most commonly used HBA, which is an additive for poultry feed [52, 53]. However, there is growing consensus that these features cannot be simply taken for granted, as they may be influenced by the emerging interactions between the HBA and the HBDs component, and thus they should be studied on a case‐by‐case basis for each DES composition, also in relation to the specific application in which they are involved [37, 54, 55, 56]. In the domain of synthetic chemistry, DESs have found wide application as reaction media for organic and inorganic transformations, also thanks to their ability to effectively dissolve both organic compounds and metal salts [57, 58, 59, 60]. One significant reason for their success, beyond their inherent green properties, lies in the fact that DESs often have a noninnocent behaviour towards the course of a reaction, being able to promote unconventional reactive pathways and stabilize intermediates, mainly through their extensive hydrogen bonding network and their Lewis or Brønsted acidic and basic functional groups, and even to act as reagents [57, 61, 62]. In addition to that, when involved in the synthesis of inorganic materials, DESs can exert an influence in orienting the formation of specific morphologies and nanostructures [63, 64, 65]. This is due to the fact that DESs present a significant content of neutral molecules (even over 50 mol%), directly impacting on the distribution of charged species and oriented dipoles that exist at the interface between the electrodes and the synthetic medium [66]. On these premises, the application of DESs as reaction media has all the credentials to drive the improvement of the sustainability profile of electrochemical synthesis. Beyond the mere replacement of VOCs with solvents that can generally be considered as more sustainable, DESs in electrosynthesis could directly address the specific issue related to the need to add a supporting electrolyte, since at least one of their components (usually the HBA) is a salt itself, thus ensuring the required electrical conductivity to carry on the process [39, 65, 67, 68]. Their character of active solvents, able to promote reactive pathways, even in a selective way, and increase reaction rates in mild conditions, is potentially suitable to be combined with the advantages of electrochemically driven transformations. Furthermore, the fairly wide range of compounds that can be used as DESs components allows tunability of the solvent's properties, offering access to *ad hoc* design of the reaction medium. It has been additionally conveyed by several authors that the exploration of a full composition range beyond the strictly “eutectic” one, and rather than being limited to fixed ratios of the components, would be worth pursuing in order to further enhance the versatility of DES and DES‐like solvents [67, 69, 70]. The present review aims at providing a picture of the state of the art in the application of DESs as solvents and promoters for electrochemical organic and inorganic synthesis, with a privileged attention to highlighting whether, and to what extent, this combination effectively results in an improvement of the synthetic methodology, from the closely intertwined points of view of reactivity and sustainability. The review focuses on the synthesis of organic compounds and inorganic materials, while the electrochemical synthesis of organic polymers in DESs, *i.e.*, electropolymerisation, is not covered; interested readers can find detailed information in dedicated reviews [71, 72, 73].

## 2 Electrochemical Potential Windows

**FIGURE 2:**
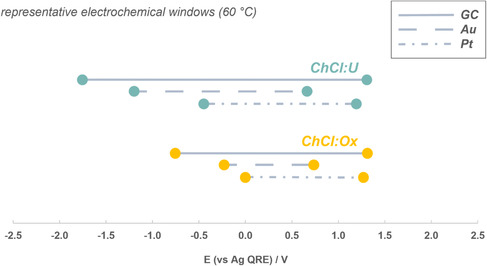
Representative ECPWs for ChCl‐based DESs at 60°C (wider for GC working electrodes) [70].

**FIGURE 3:**
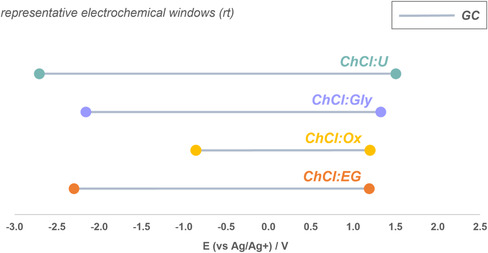
Representative ECPWs for selected ChCl‐based DESs at room temperature (width depending on the nature of HBDs) [74].

When considering DESs as potential solvents for electrosynthesis, it is crucial to determine the boundaries within which their application is technically possible, which are primarily set by the electrochemical potential window (ECPW) of the solvent, in relation to the specific working electrode material. A recent opinion paper [70] evidenced that the width of electrochemical windows for different DESs are dictated by a negative limit involving the reduction of protons (with visible oxidation of H_2_ for electrode materials possessing high hydrogen evolution reaction (HER) activity, such as Pt). This is reflected in a wider ECPW (up to 3–4 V) for DESs based on urea (U), ethylene glycol (EG), and glycerol (Gly), compared to oxalic acid (Ox, ChCl:Ox has ECPW of ∼2 V), which could be explained in terms of the different proton availability between the two HBDs (Figures 2 and 3) [70]; in addition to that, the higher content of water in hygroscopic carboxylic acids could also play a role in enhancing the HER activity. It ought to be noted that a significant aspect affecting the electrochemical behaviour of DESs is their generally high viscosity [70, 75, 76, 77, 78], which may not only hamper the suitable diffusion of the charged species [79], but also make it challenging to ensure proper stirring and processing of the reaction mixture. A strategy that could be adopted to increase the fluidity (*i.e*., decrease the viscosity) consists in performing the experiments at higher temperatures: it is therefore useful to study the ECPW of DESs at different temperatures. The data displayed in Figure 2 were recorded at 60°C [70], while in Figure 3 we highlight some of the ECPWs reported for selected ChCl‐based mixtures at room temperature, which again show a dependence of the electrochemical stability windows on the nature of different HBDs analysed [74]. In line with what was reported for Ox‐based DESs, ECPWs of around 2 V were observed for other carboxylic acid‐based DESs, by keeping ChCl as the HBA; again, intense reduction peak occurred [80]. By replacing ChCl with tetraethylammonium bromide (TEAB) as the HBA, the same trend was obtained, with TEAB:EG mixtures displaying the widest ECPW (∼3 V), while more acidic HBDs resulted in narrower boundaries, on the reductive side [80]. A detailed study showed that, with EG as the HBD, the formation of H_2_ is accompanied by decomposition pathways involving the reactivity of its primary alcohol group, resulting first in the oxidation of EG into ethylene oxide or acetaldehyde, and subsequently in the formation of a number of higher‐molecular‐weight hydroxylated esters and ethers [81]. Similarly, the secondary alcohol group in the propylene glycol (PG)‐based DES ChCl:PG 1:3 was reported to undergo oxidation into hydroxyacetone, and subsequent condensation towards higher molecular weight products [82]. In this regard, a strategy to widen the ECPW was proposed by replacing EG (and, possibly, Gly) with tertiary alcohols, such as 2,4‐dimethyl‐2,4‐pentanediol [81]. On the other side of the ECPW, positive limits are usually bound by the oxidation of chloride anions, abundant in DES mixtures (Figure 1) [70]. Indeed, the ECPW width is strongly dependent on the working electrode material, which should be thoughtfully selected when elaborating the synthetic methodology. As evident from the data reported in Figure 2, the use of a GC working electrode grants a wider ECPW for DES mixtures of different nature.

It is important to highlight that many of the syntheses herein reported were performed under galvanostatic electrolysis conditions, without assessing whether the process would take place within the ECPW of the reaction medium. Furthermore, in some cases electrolyte degradation reactions are even invoked as catalytically competent for promoting the desired transformation.

Finally, another practice that is sometimes implemented to decrease the viscosity of DESs is the addition of a controlled amount of water [76, 78, 83, 84]. With regards to the influence of such addition on the electrochemical stability, it was recently reported that the two widely employed DESs ChCl:Gly 1:2 and ChCl:EG 1:2 displayed only a modest narrowing of their ECPW, when mixed with up to 40% w/w H_2_O, thus essentially maintaining their electrochemical stability [85]. As a general observation, it has been highlighted that the electrochemical windows of DESs, although smaller than what is achieved in ILs, are wide enough for a variety of applications, including metal electrodeposition [74].

## 3 Electrosynthesis of Organic Compounds

The use of DESs as reaction media for organic transformations under electrochemical conditions is a rather recent field of investigation, with most of the literature being concentrated in the 2020s. Notwithstanding, a number of diverse synthetic methodologies have been reported, exploring the synthesis of various molecular scaffolds. In the present section, they are grouped on the basis of reaction classes and summarized in Table 1. From the point of view of the electrochemical setup, all the methodologies presented were carried on in an undivided cell configuration, except one. The working electrode is highlighted in each scheme. It should be noted that using an undivided cell may introduce possible undesired reactivity of the product, the starting materials, and the DES at the counterelectrode, while separated compartments would avoid this issue. On the other hand, in some cases concurrent anodic and cathodic processes may be functional to the desired reaction pathway.

### 3.1 Condensation Reactions

**FIGURE 4:**
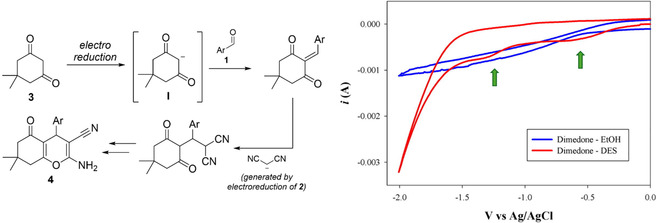
Proposed mechanism for the electrosynthesis of tetrahydrobenzo[*b*]pyrans in DES (left). Cyclic voltammetry of dimedone **3** (right) in ethanol (blue line) and in ChCl:EG DES (red line); green arrows indicate peaks attributed to electroreduction of **3** into **I**) [86]. Adapted from ref. [86] with permission. 2022, Elsevier.

**SCHEME 1:**
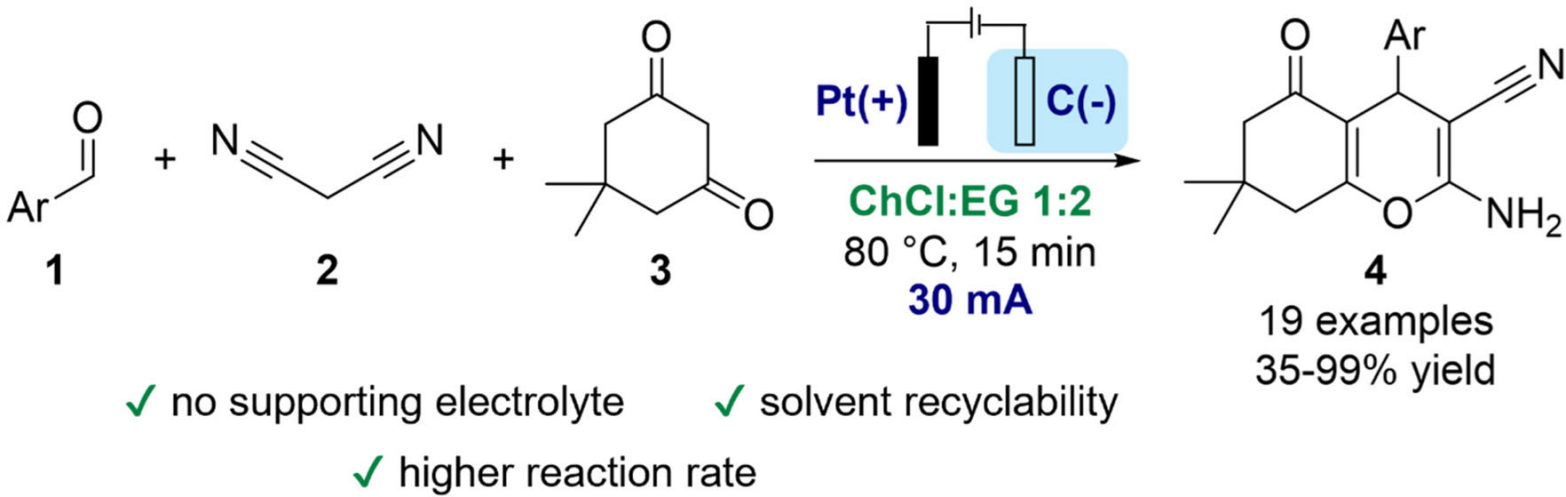
Electrosynthesis of tetrahydrobenzo[*b*]pyrans in ChCl:EG DES [86].

A multicomponent process, involving a tandem Knoevenagel condensation/Michael addition sequence, was studied in the synthesis of tetrahydrobenzo[*b*]pyrans **4**, a class of densely functionalized fused bicycles of pharmaceutical interest, using ChCl:EG 1:2 as the solvent (Scheme 1 and Table 1, entry 1) [86]. A comparative analysis with the reaction performed in conventional solvents under electrochemical conditions, or in DES under thermal conditions, revealed that the use of DES not only avoided the addition of a supporting electrolyte, being the solvent itself suitable for this role, but also increased the reaction rate. To rationalize this effect, the authors performed a cyclic voltammetry study, which suggested that, in contrast to the reaction performed in ethanol, a more efficient electrocatalytic pathway was active in DES, involving the electrochemical reduction of dimedone **3** into its conjugated base **I**, that acts as the active species undergoing condensation with aldehyde **1** (Figure 4). However, since cyclic voltammograms of the blank solvent were not provided in the paper, and based on previous literature on this topic [104, 105, 106], it could not be ruled out that the conjugated base **I** would actually be generated *via* reduction of the HBD component of the solvent (EG) and subsequent deprotonation of **3**. Deeper investigation would be needed to provide stronger mechanistic evidence. From the sustainability point of view, notably the solvent could be reused in up to three reaction cycles with only a moderate loss in the product yield (from 97% at the first cycle to 79% in the third cycle). To corroborate this improvement, compared to the methodology performed in ethanol, the authors calculated the mass‐related green metrics [9, 107], such as the E‐factor, the reaction mass intensity (RMI), and the reaction mass efficiency; however, the impressive values reported are strongly biased by the inclusion of the recovered DES in the calculations as a “product” [86]. This approach is not in line with the way recovered solvents are normally treated in green metrics determination [108, 109, 110], and more rigorous calculations would probably be required.

**SCHEME 2:**
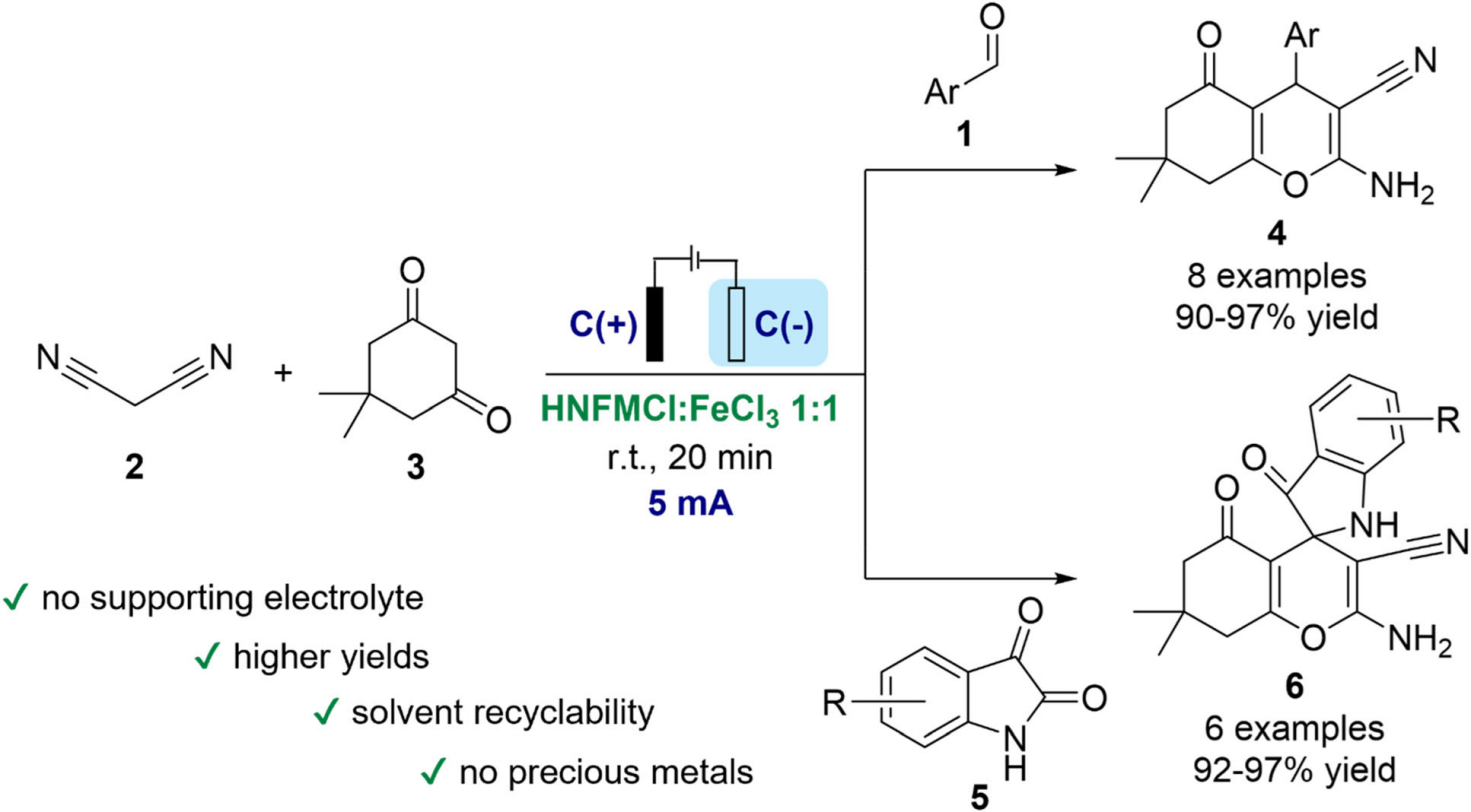
Electrosynthesis of tetrahydrobenzo[*b*]pyrans and spirooxindoles in HNFMCl:FeCl_3_ DES. The DES is prepared using FeCl_3_ · 6H_2_O and then evaporating excess water, thus the actual composition of the DES may include an undetermined amount of water [87].

The same reaction was later studied in a metal‐based DES, composed of *N*‐formylmorpholinium chloride (HNFMCl) and FeCl_3_ · 6H_2_O in 1:1 molar ratio (Scheme 2 and Table 1, entry 2) [87]. This mixture falls into the definition of “type II DES” proposed by Abbott [111], and was previously developed as an active solvent‐catalyst system for the desulfurization of fuels [112]. The authors invoked the formation of intermediate **I** (Figure 4), which was proposed to be indirectly generated as a consequence of anodic processes, that would however require further mechanistic investigation to be substantiated. Additionally, the authors speculated an effect of the Fe centre in the electrophilic activation of aldehydes **1**. This resulted in an efficient conversion of the substrates at room temperature, with reaction yields comparable to or higher than those obtained using conventional solvents, in the absence of a supporting electrolyte additive, and employing graphite as the electrodes’ material instead of precious metals such as Pt. The authors parallelly explored an analogous transformation, by replacing benzaldehydes **1** with isatins **5**, thus obtaining spirooxindoles **6** (Scheme 2). This reaction was also used as a model to verify the recyclability of the solvent, which could be reused up to 8 cycles without a significant decrease in the product yield. Both classes of products, **4** and **6**, were tested for their potential anticancer activity, by evaluating their in vitro cytotoxicity against two lines of cancer cells [87].

**SCHEME 3:**
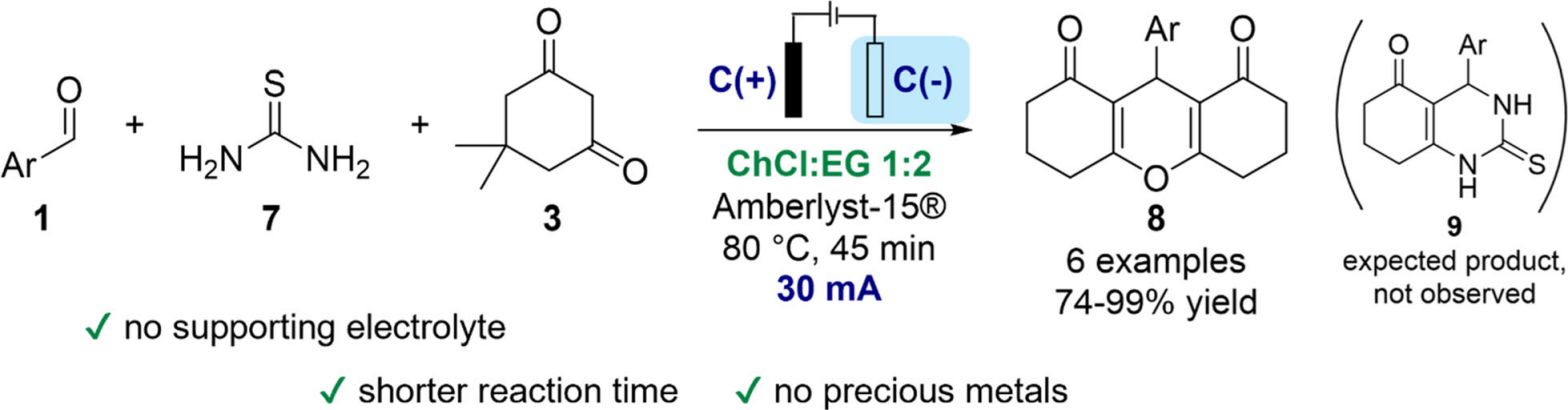
Electrosynthesis of tetrahydroxanthenediones in ChCl:EG DES [88].

In the domain of multicomponent reactions, the Biginelli reaction could be in principle performed using the same reagents as in Scheme 1, by replacing malononitrile **2** with thiourea **7** (Scheme 3 and Table 1, entry 3) [113, 114]. Envisaging a catalytic effect similar to the previously reported case (Scheme 1 and Figure 4), the same authors attempted the transformation in the DES ChCl:EG 1:2, in the presence of the heterogeneous acid catalyst Amberlyst‐15. Unexpectedly, they found that **7** was not incorporated into the product to afford the Biginelli product **9**, as the condensation between **1** and **3** alone resulted instead in the highly selective formation of tetrahydroxanthenediones **8** (Scheme 3) [88]. Encouraged by this serendipitous outcome, the authors explored the synthesis of these symmetrical molecular scaffolds, highlighting that the use of the ChCl:EG DES as solvent enabled shorter reaction times than those previously reported under electrochemical conditions in conventional solvents [113, 114], and no supporting electrolyte was required [88].

**SCHEME 4:**
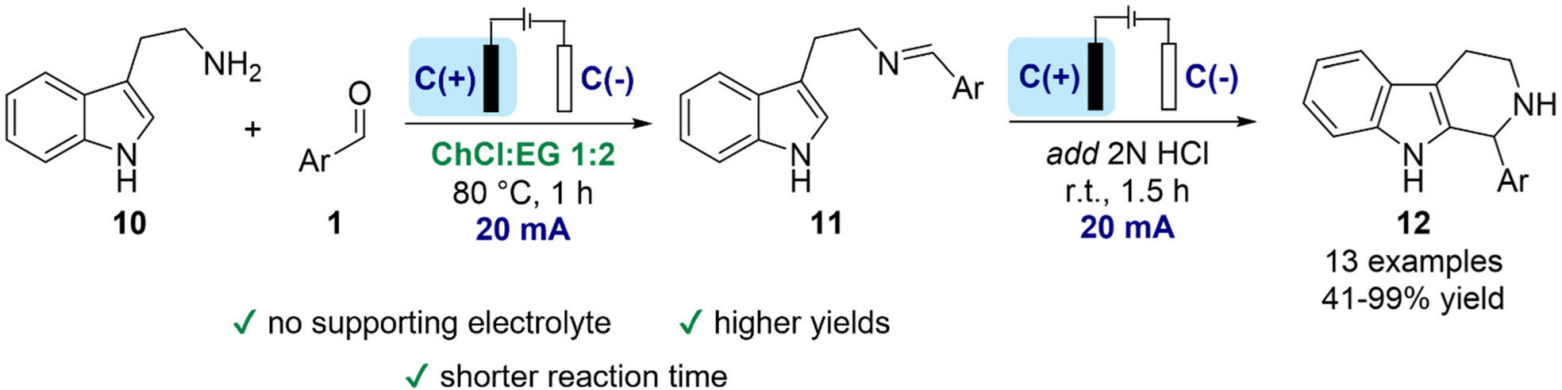
Electrosynthesis of tetrahydro‐β‐carbolines in ChCl:EG DES [89].

Similar reaction conditions were also exploited to synthesize tetrahydro‐β‐carboline derivatives **12** through the Pictet–Spengler reaction, which consists in the condensation between tryptamine **10** and aromatic aldehydes **1**, followed by a tandem Friedel–Crafts‐type arylation/1,2‐alkyl shift of the intermediate Schiff base **11** (Scheme 4 and Table 1, entry 4) [89]. Compared to previously reported methodologies for the synthesis of these molecular scaffolds [115, 116], the combination of the ChCl:EG 1:2 DES and the electrochemical conditions significantly improved the reaction rates and the overall yield of the process. The authors attributed this synergistic effect to a hydrogen‐bonding activation of substrates **10** and **1** by the DES components. Furthermore, a significant improvement was observed in the efficiency of the second step, i.e., the conversion of the Schiff base **11** into the final product **12**, which was attributed to a superior electrocatalytic efficiency for the oxidative activation of **11**, postulated on the basis of a cyclic voltammetry study [89]. However, it looks like deeper investigation would be needed to ascertain the electrochemical influence on the reaction mechanism with a sufficient degree of certainty.

**FIGURE 5:**
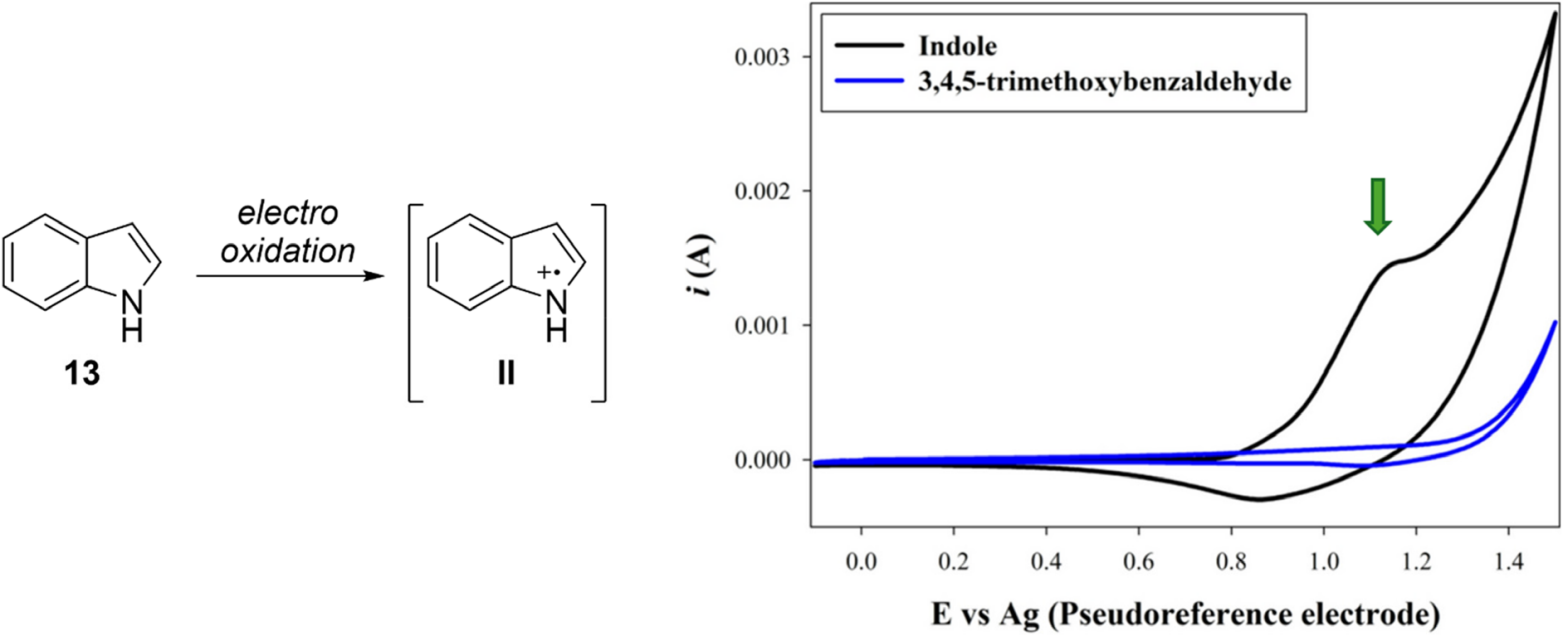
Proposed key intermediate in the synthesis of bis(indolyl)methanes in DES (left). Cyclic voltammetry of indole **13** and benzaldehyde **1** (right), adapted from ref. [90] (licensed under CC‐BY 4.0, 2024, Springer Nature). The green arrow indicate the peak attributed to electrooxidation of **13** into **II** [90].

**SCHEME 5:**
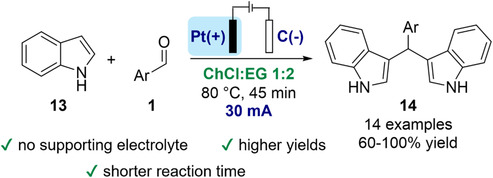
Electrosynthesis of bis(indolyl)methanes in ChCl:EG DES [90].

Analogous results were also obtained in another synthesis of pharmaceutically active compounds, i.e., bis(indolyl)methanes **14** (Scheme 5 and Table 1, entry 5) [90]. Here, again, the reaction performed in DES and under electrochemical conditions attained higher yields and lower times than previously reported methods [117], and did not require the addition of a supporting electrolyte. Based on cyclic voltammetry, the reaction was credited to proceed through electrooxidation of indole **13** into intermediate **II** (Figure 5). However, no hypotheses were put forward to explain a possible active effect of the DES in the mechanism, as no comparative electrochemical studies in other solvents were carried out [90].

### 3.2 Organometallic Nucleophilic Addition Reactions

**SCHEME 6:**
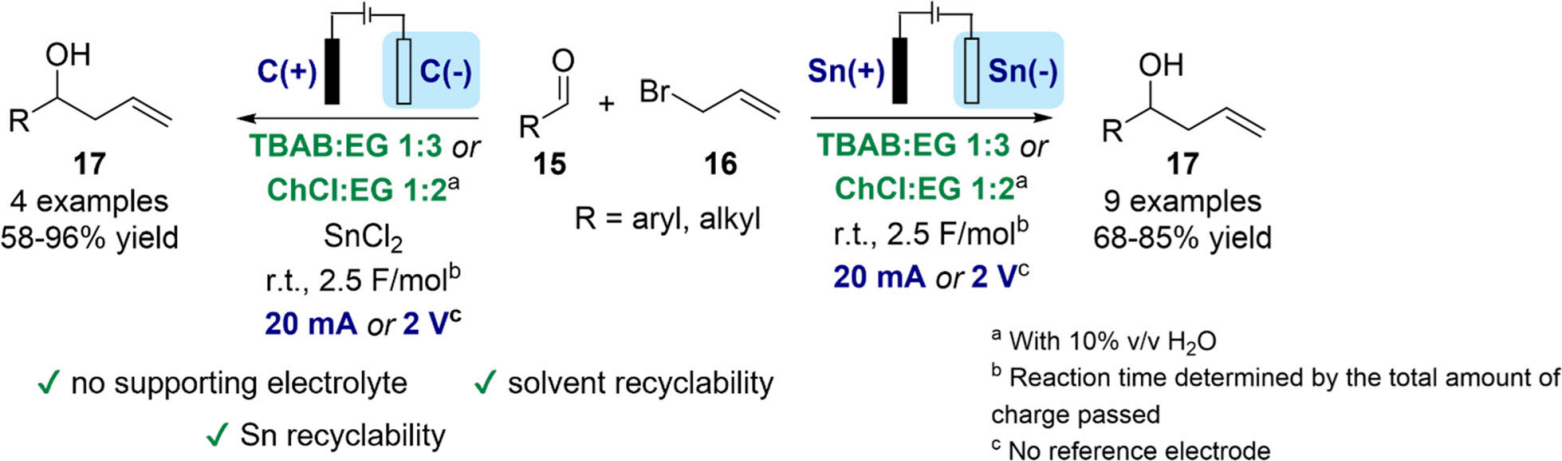
Electrosynthesis of allylic alcohols in TBAB:EG or ChCl:EG DES [91].

Another interesting class of organic transformations that has been studied by taking advantage of electrochemical synthesis in DESs is represented by addition reactions. The electroreduction of allyl bromide **16** in the presence of a Sn source, using either TBAB:EG 1:3 or ChCl:EG 1:2 as the solvent, allowed to produce the allylic organometallic species able to react with aldehydes **15**, eventually affording differently substituted secondary alcohols **17** by nucleophilic allylation (Scheme 6 and Table 1, entry 6) [91]. The exact nature of the active allyl tin intermediate was not investigated, although the literature suggests that an equilibrium between monomeric and dimeric allyl tin mixed halides could take place [118, 119]. As a source of Sn, either a sacrificial Sn electrode, or externally added SnCl_2_, coupled with graphite electrodes, could be used. Apart from the possibility of running the reaction in the absence of a supporting electrolyte, and of recycling the solvent system for more than one reaction cycle, as discussed also for previous examples, a major sustainability improvement was here represented by the electrochemical recycling of the reducing metal from the reaction mixture. This was made possible since DESs are well known solvents for electrodeposition of various metals, *vide infra* [120]. The authors demonstrated the viable recovery of up to 99% of the initial Sn amount, when the reaction was performed in the presence of SnCl_2_, in the form of metallic Sn deposited onto the graphite electrode, by simple electrolysis of the reaction mixture, after removal of the organic product through extraction. The recovered Sn was then successfully employed in an additional allylation cycle [91].

**SCHEME 7:**
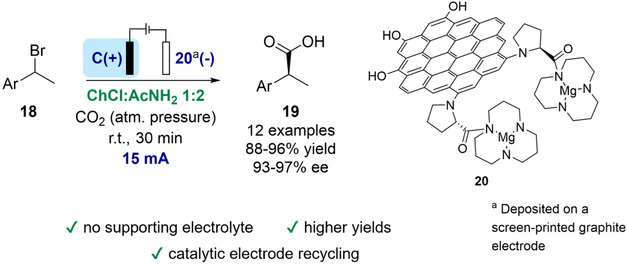
Asymmetric electrosynthesis of carboxylic acids by Grignard reaction in ChCl:AcNH_2_ DES. Mg is present mainly as Mg NPs [92].

**SCHEME 8:**
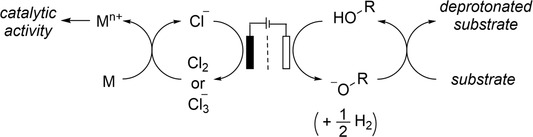
Electrocatalytic generation of active species from the DES components, and their activity in the reactions presented in this Section. Chloride ion is provided by ChCl, while R‐OH is the HBD (usually EG); M = metal (Cu, Fe, Mg, etc*.*) [70, 122].

In the domain of electrocatalysed additions of organometallic compounds to electrophiles, some studies have focused on the incorporation of a carboxylic acid moiety into organic substrates, by means of nucleophilic addition to CO_2_. This transformation is particularly appealing because it is functional to the synthesis of pharmaceutically active compounds, in which the carboxylic group is frequently found [121], through the repurposing of one of the most environmentally problematic molecules of the contemporary age, carbon dioxide. In particular, (1‐bromoethyl)benzenes **18** were used as substrates to produce the corresponding Grignard reagent, which acted as a nucleophile in the addition to CO_2_, eventually resulting in the carboxylic derivatives **19** (Scheme 7 and Table 1, entry 7) [92]. The reaction was performed in the DES ChCl:AcNH_2_ 1:2 as the solvent, and took advantage of a Mg‐based chirally modified cathode, obtained by depositing graphene oxide **20**, functionalized with a Mg‐coordinating decaline ligand through a chiral L‐proline linker (Scheme 7), onto a graphite screen‐printed electrode. In the proposed catalytic mechanism, the key electrochemical event does not take place at the modified cathode, but rather it is the anodic oxidation of Cl^−^ into Cl_2_: indeed, the characterization of **20** revealed that Mg was mainly present as Mg NPs, which required oxidation from Cl_2_ to produce the active Mg species, able to convert **18** into the analogous organomagnesium reagent, thus initiating the Grignard mechanism [92]. The electrochemical decomposition of Cl^−^ into Cl_2_ in DESs had already been investigated, and the results had pointed towards the prevalence of dissolved Cl_3_^−^, rather than gaseous Cl_2_, as the predominant form in these systems (Scheme 8) [70, 122]. It ought to be noted that the other mechanistic steps proposed in the paper would actually need to be corroborated by stronger experimental evidence, and must thus be seen as a tentative interpretation of the reactivity. Notably, carboxylic acids **19** were obtained in high yields and enantiomeric purities, and comparative experiments indicated the superiority of the DES over VOCs. Importantly, the active electrode (but not the solvent) could be recycled several times, reducing the production of waste [92].

**SCHEME 9:**
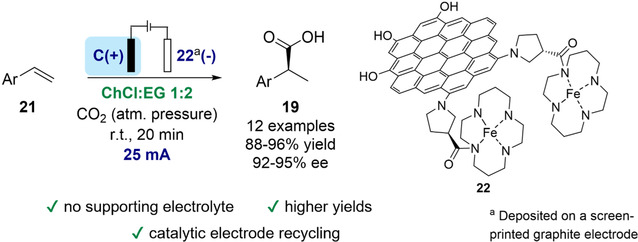
Asymmetric electrosynthesis of carboxylic acids by reaction of styrenes and CO_2_ in ChCl:EG DES. Fe is present mainly as Fe NPs [93].

The enantioselective synthesis of carboxylic acids **19** with CO_2_ was also achieved starting from styrene derivatives **21**, in ChCl:EG 1:2 as solvent (Scheme 9 and Table 1, entry 8) [93]. The methodology was analogous to the one described for the Grignard reaction, with some modifications: graphene oxide was functionalized with (S)‐pyrrolidine‐3‐carboxylic acid as linker and 1,4,8,11‐tetraazacyclotetradecane as ligand, and the metal centre was Fe (Scheme 8). As in the previous study, the DES components were indicated to actively take part in the electrocatalysed process, through the production of a catalytic amount of Cl_2_, from ChCl, involved in the formation of the Fe active species, which would activate both the CO_2_ molecule and the styrene moiety of **21**. Additionally, the electrochemical reduction of EG into 2‐hydroxyethanolate would result in the formation of the nucleophile that eventually reacts with the activated CO_2_ (Scheme 8). However, no strong mechanistic evidence was provided to corroborate the proposed reaction pathway. The reaction performed in DES afforded higher yields than conventional solvents, and the functionalized electrode could be used for ten cycles with only a modest decrease in the yield of product **19** [93].

**SCHEME 10:**
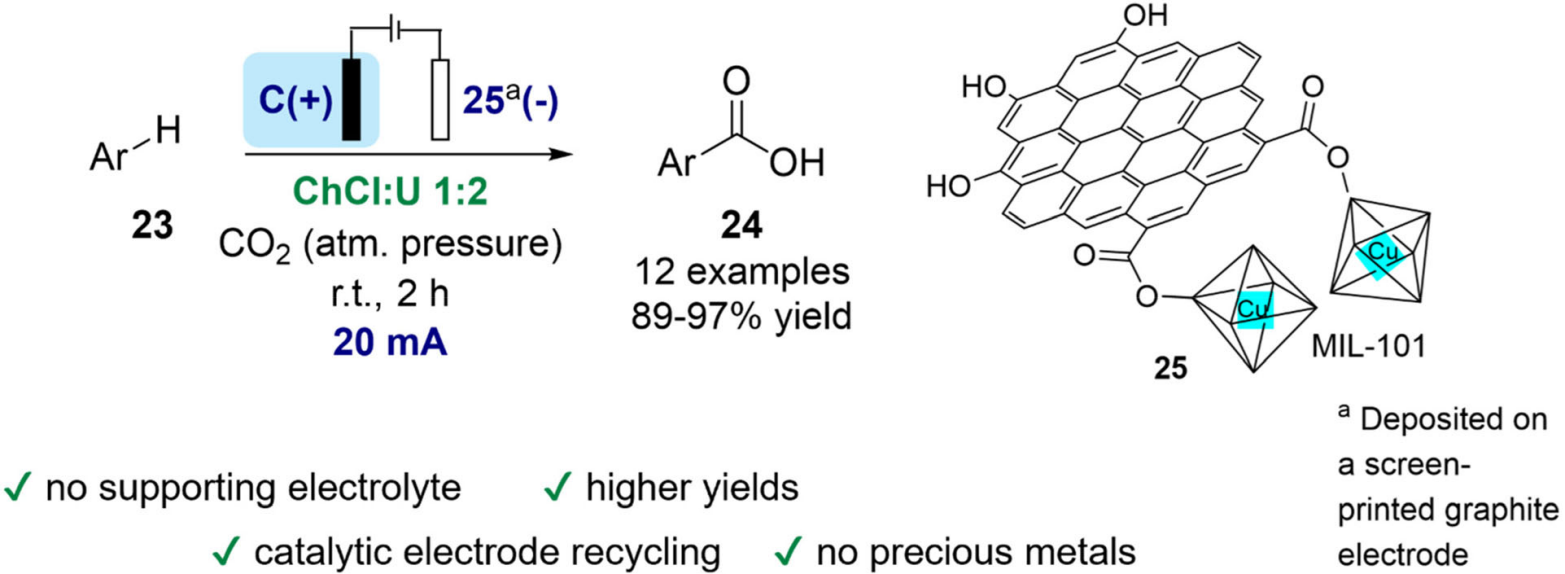
Electrosynthesis of carboxylic acids by Kolbe–Schmitt reaction in ChCl:U DES [94].

Another carboxylation reaction, exploiting the addition to CO_2_ as an electrophile, was reported with a Cu‐promoted Kolbe–Schmitt‐type reaction, an electrophilic aromatic substitution of benzene and naphthalene derivatives **23**, which afforded benzoic or naphthoic acids **24** (Scheme 10 and Table 1, entry 9) [94]. In this case, the active electrode was prepared by grafting the MIL‐101 metal–organic framework (MOF) [123] onto graphene oxide, including Cu NPs into its porous structure, and subsequently coating the material on a screen‐printed graphite electrode. The only DES tested in this work was ChCl:U 1:2, which avoided the use of an additional supporting electrolyte and afforded considerably higher yields, in the comparative study with conventional solvents. This was attributed to a better stabilization of charged intermediates in the ionic DES environment; furthermore, the DES components were hypothesized to actively take part in the catalytic cycle, by generating the active Cu species from the Cu NPs (Scheme 8). It ought to be noted that employing a Cu‐based catalyst represented a substantial sustainability improvement, compared to previously reported electrochemical methodologies that instead relied on nonabundant noble metals (mainly Pt and Ag); moreover, embedding the metal centre in the porous MOF structure was highly suitable for the CO_2_ capture and utilization. The active electrode could be recycled up to 11 times, before observing a slight reduction in the carboxylation yield [94].

### 3.3 Miscellaneous Reactions

**SCHEME 11:**
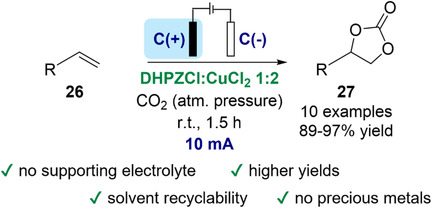
Electrosynthesis of cyclic carbonates by oxidation and carboxylation of alkenes reaction in DHPZCl:CuCl_2_ DES. Reaction performed in the presence of atmospheric O_2_ [95].

**SCHEME 12:**
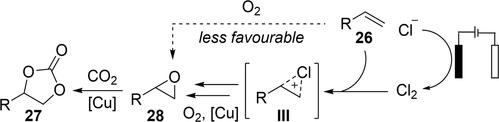
Proposed pathways in the electrosynthesis of cyclic carbonates in DHPZCl:CuCl_2_ DES [95].

The potential of DESs as designer solvents in the electrochemically promoted incorporation of CO_2_ into organic scaffolds was further evidenced by the conversion of olefins **26** into cyclic carbonates **27** (Scheme 11 and Table 1, entry 10) [95], where a DES, obtained by combining 1,4‐bis(2,3‐dihydroxypropyl)piperazine dihydrochloride (DHPZCl, see Figure 1 for the molecular structure) and CuCl_2_ in 1:2 molar ratio, played the double role of electrolyte and catalyst. The reaction was performed using graphite as the electrode material, affording higher yields in DES than in conventional electrolytes, and the active solvent could be recycled up to 9 times, maintaining effectiveness in promoting the conversion of **26** into **27**. A computational study on the reaction mechanism suggested a key catalytic role of Cl_2_, generated by anodic oxidation of Cl^−^, in providing access to a favourable pathway involving the formation of the chloronium intermediate **III**, which is then supposed to be converted into epoxide **28** by Cu‐promoted reaction with atmospheric O_2_; instead, in the absence of Cl_2_, the direct oxidation of **26** into **28** turned out to have a significantly higher energy barrier (Scheme 12). The epoxide **28** is then credited to incorporate the CO_2_ molecule, with the assistance of the Cu catalytic centre, eventually affording the final product **27**. However, it ought to be mentioned that no experimental details on the computational methods used for the calculations were provided in the paper. Furthermore, the author described **III** as an intermediate, but treated it as a transition state in their proposed reaction profile, thereby leaving some ambiguity on the nature of the kinetic barriers reported [95].

**SCHEME 13:**
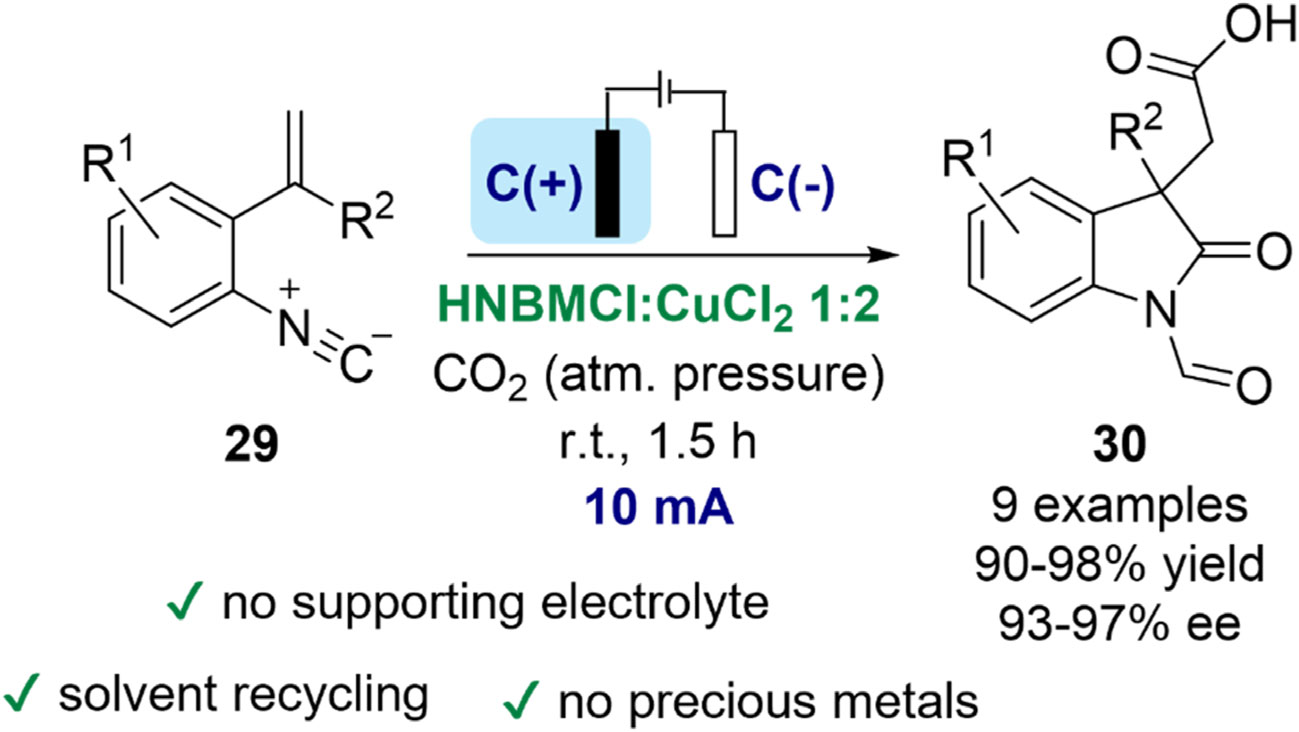
Electrosynthesis of (R)‐2‐(1‐formyl‐3‐aryl‐2‐oxoindolin‐3‐yl)acetic acids in HNBMCl:CuCl_2_ DES [96].

An example of the construction of a complex molecular architecture, involving the incorporation of CO_2_, was reported with the asymmetric synthesis of (R)‐2‐(1‐formyl‐3‐aryl‐2‐oxoindolin‐3‐yl)acetic acids **30** from substituted 1‐isocyano‐2‐(1‐phenylvinyl)benzenes **29** (Scheme 13 and Table 1, entry 11) [96]. The reaction was performed using an *ad hoc* DES, in which the HBA was the chiral salt (S)‐*N*‐(2‐methylbutanoyl)morpholinium chloride (HNBMCl, see Figure 1 for the structure), and CuCl_2_ played the role of the HBD. The authors invoked a chirality transfer from the HBA to the substrate, accounting for the high enantiomeric excesses observed in products **30** (Scheme 13). This point would require more experimental work to be unambiguously clarified: up to now, DES components have been reported to effectively influence the stereochemical outcome of a reaction only in those cases where they are covalently bound to the substrate in the chirality‐defining step or, in other words, when they act as organocatalysts [124, 125, 126], which does not seem to be the case here. Moreover, from the mechanistic point of view, the authors claimed the electrooxidation of Cl^−^ into Cl_2_, and subsequent reaction with the substrate's double bond, as the key event initiating the transformation, through a chloronium intermediate analogous to intermediate **III** in Scheme 5. To prove the presence of Cl_2_, a I_2_/Na_2_S_2_O_3_ titration of the reaction mixture was performed; however, only its qualitative observation was reported, and a comparison with the same titration performed on the blank solvent (with or without applied current) would be needed to provide a clearer picture of the process. Notably, the DES system could be recycled to up to 9 reaction runs, using graphite as both anode and cathode; however, the preparation of the HBA required two synthetic steps, one of which involved the use of the hazardous reagent SOCl_2_, thus somehow losing one of the main sustainable features of DESs, that is their straightforward preparation by simply mixing two or more widely available components [96].

**SCHEME 14:**
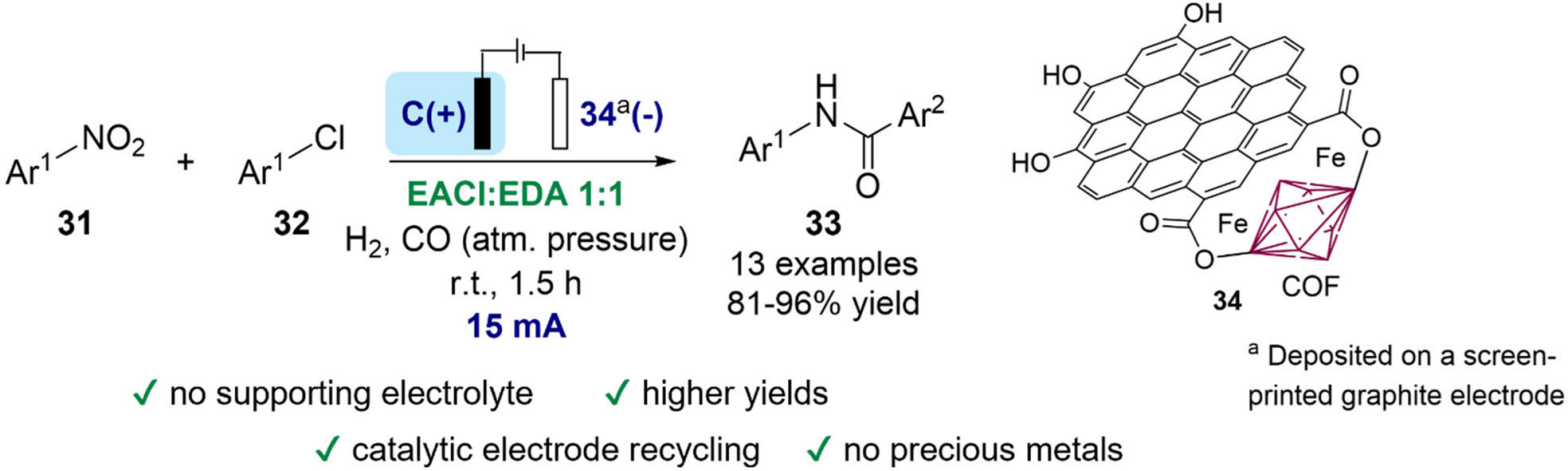
Electrosynthesis of *N*‐phenylbenzamides by tandem reduction/carbonylation/arylation of nitrobenzenes with chlorobenzenes in EACl:EDA DES. The authors did not specify whether the Fe centres are coordinated cations or Fe NPs [97].

A recent paper reported on a DES‐based electrocatalytic methodology to synthesize substituted *N*‐arylbenzamides **33** through a tandem process involving hydrogenation of nitrobenzenes **31**, followed by carbonylation with carbon monoxide and functionalization with chlorobenzenes **32** (Scheme 14 and Table 1, entry 12) [97]. The active material for the cathode was here obtained by modification of graphene oxide with Fe sites and an amine‐rich covalent organic framework (COF), able to efficiently adsorb H_2_ and CO in its porous structure. Upon testing different VOCs, with NaCl as the supporting electrolyte, and DESs in the absence of a supporting electrolyte, the best performances were observed when carrying out the reaction in a DES composed by ethanolamine hydrochloride (EACl) and ethylenediamine (EDA) in 1:1 molar ratio. The effectiveness of this specific DES was attributed to its ability to increase CO capture *via* carbonylation of the amine functional groups in the solvent's molecular structure, thus highlighting once again the importance of an *ad hoc* design of the reaction medium. Nonetheless, the reaction mechanism proposed is likely to be considered as a tentative one, since no experimental results were yet provided to support it. A comparative study of the reaction conditions revealed a synergistic effect of the simultaneous presence of Fe, COF, DES, and electric current, resulting in a high‐yielding, noble metal‐free methodology. Additionally, the system could be reused up to 9 cycles, with only a moderate loss in the yield of **33** [97].

**SCHEME 15:**
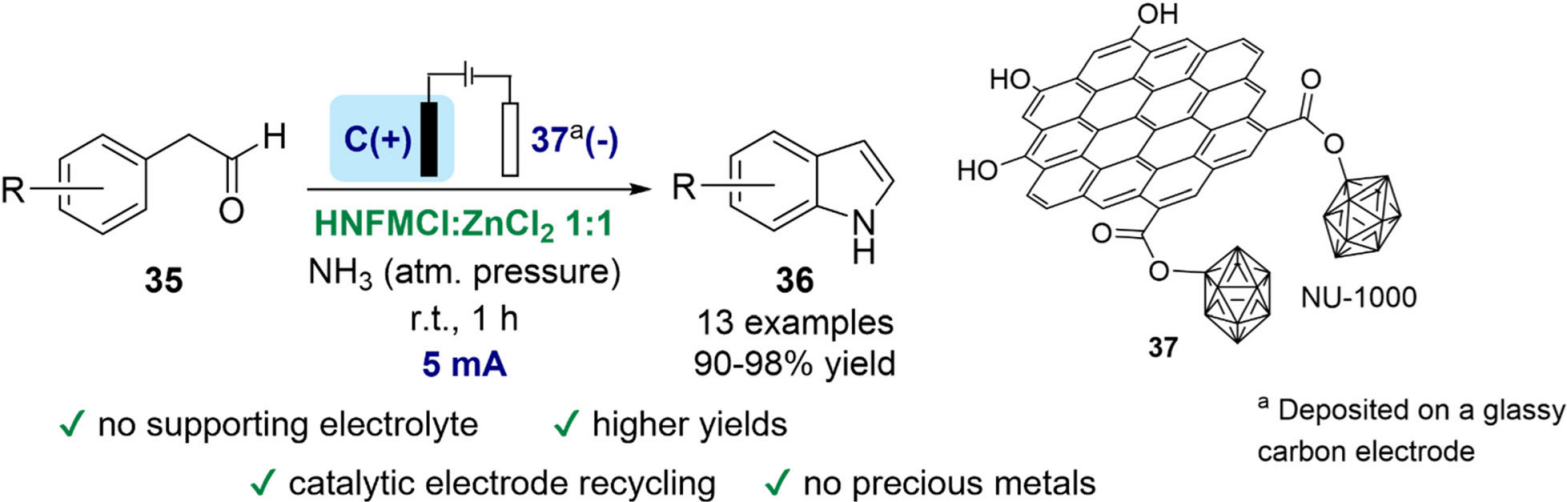
Electrosynthesis of indoles by amination/cyclization of phenylacetaldehydes with NH_3_ in HNFMCl:ZnCl_2_ DES [98].

The strategy of modifying the cathode with a porous material to enhance reactive gas absorption was exploited also in the synthesis of indoles **36** from phenylacetaldehydes **35** (Scheme 15 and Table 1, entry 13), where the NU‐1000 MOF [127], grafted onto graphene oxide, allowed for the efficient capture and utilization of gaseous NH_3_ [98]. The reaction was performed in the HNFMCl:ZnCl_2_ 1:1 DES, analogous to the one used for the multicomponent reactions reported in Scheme 2. The reaction is supposed to take place through amination of the aldehyde functional group in **35**, followed by ring closure. The solvent, which again allowed the electrochemical synthesis to take place in the absence of any supporting electrolyte, was reported to play an active role in the catalytic cycle, by facilitating the indole ring closure *via* insertion of Zn on the C–H bond. Furthermore, it was speculated that electrogenerated Cl_2_ (Scheme 8) could play a role in assisting the cleavage of Zn to release the final product [98]. However, no experimental data were included to support this claim. High yields of indoles **36** were obtained, in the absence of any precious metal in the electrode or in the catalytic system, and with the possibility to run the reaction up to 9 cycles. The obtained indole scaffolds were also tested as potential anticancer active pharmaceutical ingredients [98].

**SCHEME 16:**
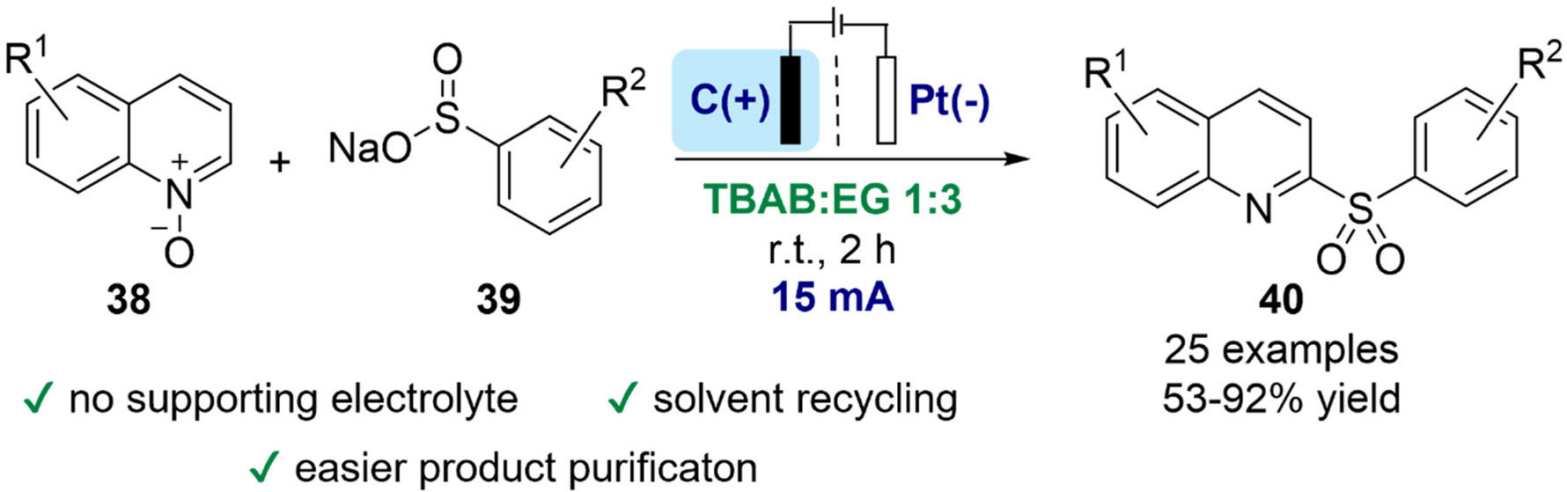
Electrosynthesis of diaryl sulfones by deoxygenative sulfonylation TBAB:EG DES [128].

**SCHEME 17:**
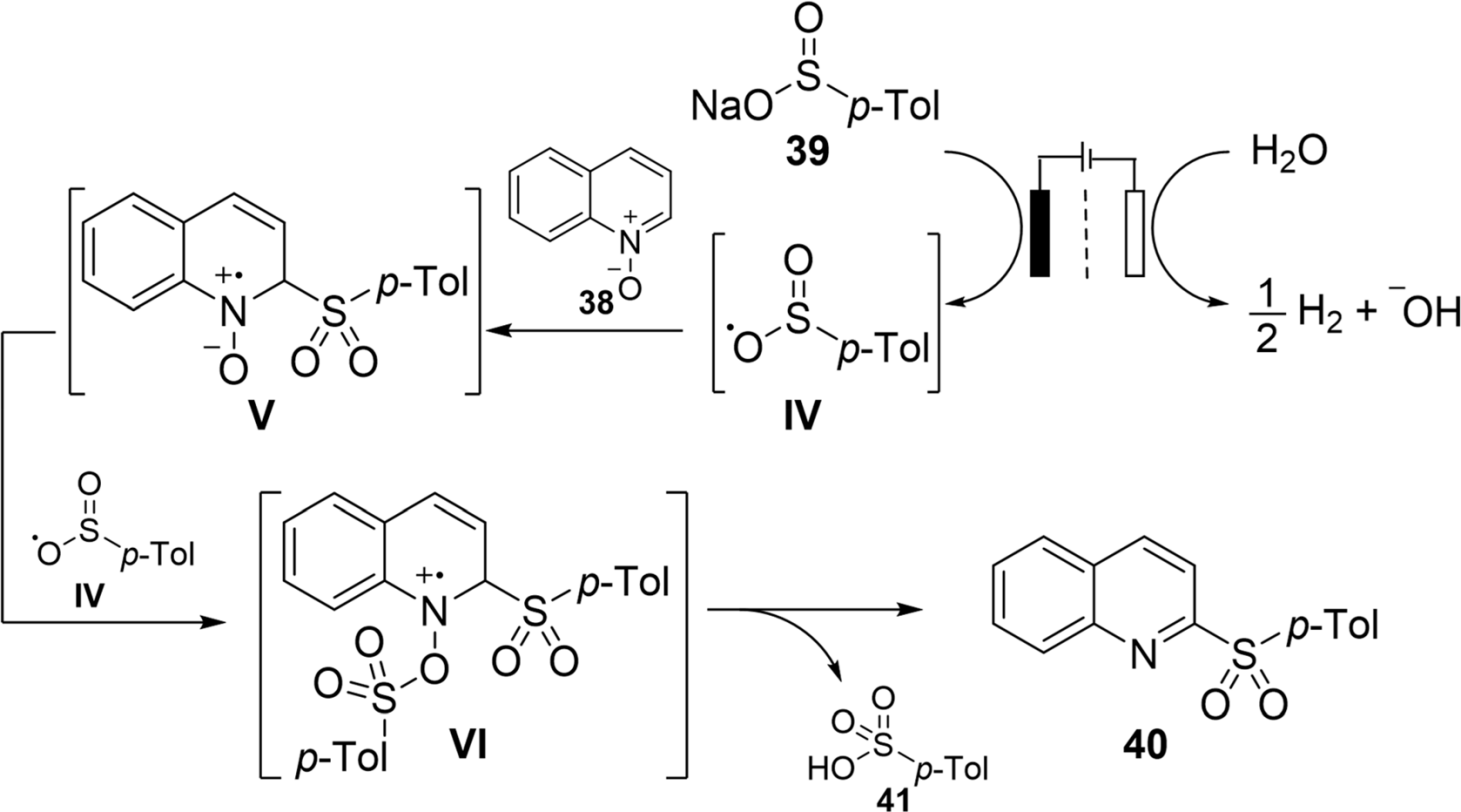
Proposed mechanism for the synthesis of diaryl sulfones. *p*‐Tol = *para*‐tolyl [128].

The electrochemical deoxygenative sulfonylation of quinoline *N*‐oxides **38** with aryl sulfinates **39**, to obtain diaryl sulfones **40**, was reported using the DES TBAB:EG 1:3 as medium (Scheme 16 and Table 1, entry 14) [128]. Interestingly, this is, to the best of our knowledge, the only organic electrosynthetic methodology that employed a divided cell configuration: indeed, the authors reported that the reaction did not afford the desired product in an undivided cell. The mechanism, also based on previous studies performed in acetonitrile [99], was credited to involve the anodic oxidation of **39** into radical intermediate **IV**, which undergoes selective arylation on the *ortho* position of **38**. The so‐formed intermediate **V** reacts with a second equivalent of **IV**, finally affording the final product **40** through release of *p*‐toluenesulfonic acid **41** (Scheme 17). In the cathodic compartment, HER from an aqueous NaCl solution takes place. The main advantage over the previous methodology in a conventional solvent [99], apart from eliminating the need for any supporting electrolyte, consisted in the possibility of easily recovering and purifying the product, by simply precipitating it upon water addition. This was reflected in a significant improvement in the mass‐related *green* metrics, such as the E‐factor and the RMI. On the other hand, no specific active effects of the DES on the chemical transformation could be highlighted: cyclic voltammetry of **39** in DES was performed, but it would be helpful to evaluate the solvent effect on its oxidation potential through a comparison in non‐DES solvents/systems. Finally, the authors showed the feasibility of recycling the solvent system, which could be used for 5 consecutive runs with only a modest reduction in the yield of **40** [128].

### 3.4 Biomass Valorisation Reactions

**SCHEME 18:**
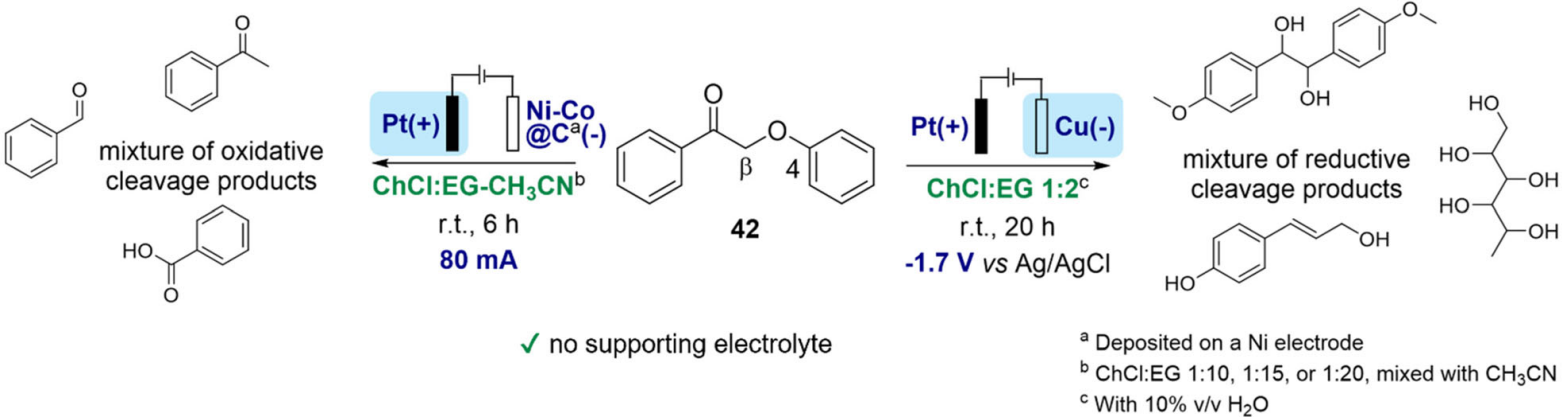
Electrochemical oxidative [101] and reductive [100] cleavage of 2‐phenoxyacetophenone as model compound for lignin β‐O‐4 motif. Some of the main products are herein shown.

While all the organic electrochemical methodologies in DES introduced so far involve the construction of molecular complexity, with a privileged view to the application in pharmaceutical industry, some research efforts have also been dedicated to investigating the electrocatalytic cleavage of 2‐phenoxyacetophenone **42**. The reaction is of interest to the production of platform chemicals from biomass, specifically in the depolymerisation of lignin, since **42** represents a model compound for the lignin β‐O‐4 motif (Scheme 18 and Table 1, entries 15–16). The cleavage was achieved under both reductive [100] and oxidative [101] conditions, using EG‐based DESs as solvents. In this case, the purpose of employing a DES was essentially in view of the actual application in lignin depolymerisation, as these solvents are known for their ability to effectively solubilize the biopolymer [129, 130, 131]. However, some of the products obtained in the electroreductive cleavage of **42** showed the incorporation of EG, or EG‐derived fragments, into their structure, suggesting an active involvement of the DES in the reaction mechanism (Scheme 13) [100]. Apart from that, no other significant advantages of combining DES with electrochemical conditions were reported, except, again, the absence of any supporting electrolyte. It should also be noted that, for the oxidative cleavage work, the “DESs” were composed of ChCl and EG in 1:10, 1:15, and 1:20 molar ratio, which are actually far from the eutectic composition [132, 133], and should perhaps be more properly defined as solutions of ChCl in EG; furthermore, an unspecified amount of acetonitrile, a conventional organic solvent, was in turn added to these mixtures for the electrocatalytic experiments [101]. On these bases, we can consider this process as a borderline, even if effective, example compared to those rigorously using a DES‐based electrolyte.

**SCHEME 19:**
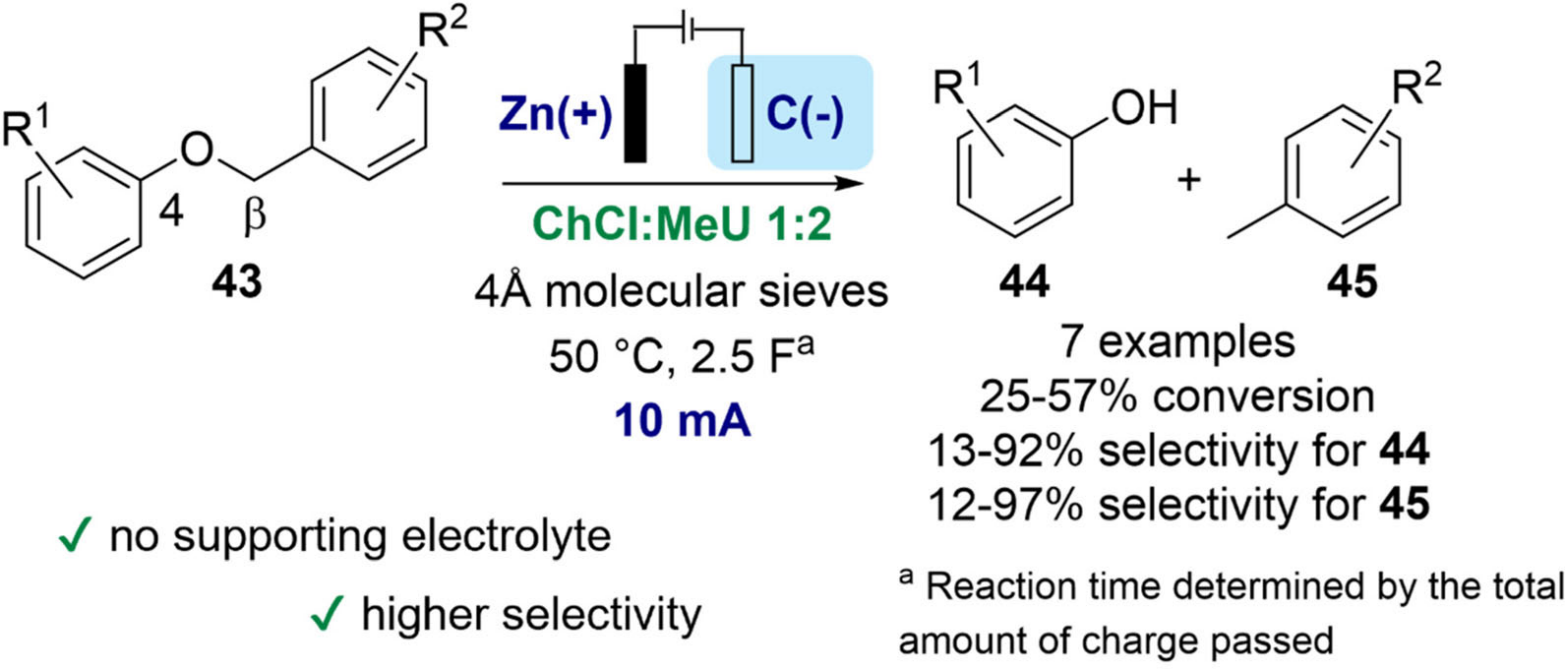
Electrochemical reductive cleavage of benzyl phenyl ethers as model compounds for lignin β‐O‐4 motif [102].

The electrochemical reductive cleavage of the lignin β‐O‐4 motif in DESs was recently investigated on another model compound, that is benzyl phenyl ether and its substituted derivatives **43** (Scheme 19 and Table 1, entry 17) [102]. The study focused on the formation of phenols **44** and toluenes **45** as target products, thus taking into account the total conversion of **43**, and the yield of these two products among all others. Extensive investigation on the influence of the reaction parameters, such as the nature of the DES, the working electrode (cathode) and the counterelectrode (anode), and the presence or absence of molecular sieves to mitigate competing HER reaction from adventitious water, was carried on. In particular, several DESs were screened as possible reaction media, in the absence of any supporting electrolyte, and their electrochemical features were characterized: it was found that the best conversions of **43** resulted from a large cathodic stability of the solvent (i.e., cathodic ECPW boundary at more negative potential), combined with a shift of the onset reduction potential of **43** towards more positive values. The most effective combination of these two parameters was identified in the 1:2 mixture of ChCl and *N*‐methylurea (MeU), which was then used as the medium for the substituent scope of the reaction. Interestingly, comparing the results in DES with those obtained by performing the reaction in a conventional solvent, *N*,*N*‐dimethylformamide (with Bu_4_NPF_6_ as supporting electrolyte), revealed that the latter afforded higher conversions of **43**, but with a lower selectivity towards the desired products **44** and **45**; this was attributed to the DES‐induced stabilisation of the products through hydrogen bonding, thus preventing their electrochemical degradation. Additionally, the authors performed a recyclability test of the DES, observing a significant decrease in the conversion of **43** (although not in the selectivity towards **44** and **45**), which was attributed to accumulation of water in the solvent structure, hypothesized *via* NMR study [102].

**SCHEME 20:**
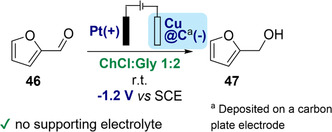
Electrochemical reductive cleavage of furfural in ChCl:EG DES [103].

In the domain of biomass conversion, the electrochemical reduction of furfural **46** to furfuryl alcohol **47** was investigated in the DES ChCl:Gly 1:2 (Scheme 20 and Table 1, entry 18) [103]. The study did not provide quantitative data on the reaction yield, but in situ Fourier‐transform infrared spectroscopy showed that the conversion of **46** into **47** took place effectively. More importantly, the work focused on two key aspects to be considered in performing an electrochemical organic reaction in eutectic mixtures, i.e., the possible degradation of the reaction substrate by interaction with the DES components, and the electrochemical stability of the solvent itself under the working conditions. On these regards, the authors highlighted the importance of the choice of the electrode material (in this case, Cu on carbon showed less DES degradation than Au on carbon) and of the DES nature (for instance, ChCl:U 1:2 was discarded because **46** underwent condensation with urea) [103].

**TABLE 1:** Summary of the utilization of DESs in organic electrosynthesis.

Entry	**DES/DES‐like** **medium**	Electrodes material	Electrolysis conditions	Transformation	Ref.
1	ChCl:EG 1:2	Pt(+)/C(−)^a^	15 mA 80°C, 15 min	Knoevenagel condensation/Michael addition	[86]
2	HNFMCl:FeCl3 1:1	C(+)^a^/C(−)^a^	5 mA r.t., 20 min	Knoevenagel condensation/Michael addition	[87]
3	ChCl:EG 1:2	C(+)^a^/C(−)^a^	30 mA 80°C, 45 min	Knoevenagel condensation	[88]
4	ChCl:EG 1:2	C(+)^a^/C(−)^a^	20 mA 80°C, 1 h then r.t., 1.5 h	Imine formation/Friedel–Crafts‐type arylation	[89]
5	ChCl:EG 1:2	Pt(+)/C(−)^a^	30 mA 80°C, 45 min	Condensation	[90]
6	TBAB:EG 1:3 orChCl:EG 1:2 +10% v/v H_2_O	C(+)^a^/C(−)^a^ *or* Sn(+)/Sn(−)	20 mA or 2V r.t., 2.5 F/mol	Grignard reaction	[91]
7	ChCl:AcNH2 1:2	C(+)^a^/**20**(−)^b^	15 mA r.t., 30 min	Asymmetric Grignard reaction	[92]
8	ChCl:EG 1:2	C(+)^a^/**22**(−)^b^	25 mA r.t., 20 min	Asymmetric Grignard reaction	[93]
9	ChCl:U 1:2	C(+)^a^/**25**(−)^b^	20 mA r.t., 2 h	Kolbe–Schmitt‐type reaction	[94]
10	DHPZCl:CuCl_2_ 1:2	C(+)^a^/C(−)^a^	10 mA r.t., 1.5 h	Synthesis of cyclic carbonates	[95]
11	HNBMCl:CuCl_2_ 1:2	C(+)^a^/C(−)^a^	10 mA r.t., 1.5 h	Synthesis of highly substituted indolinones	[96]
12	EACl:EDA 1:1	C(+)^a^/**34**(−)^b^	15 mA r.t., 1.5 h	Hydrogenation/carbonylation/arylation	[97]
13	HNFMCl:ZnCl_2_ 1:1	C(+)^a^/**37**(−)^c^	5 mA r.t., 1 h	Imine formation/Friedel–Crafts‐type arylation	[98]
14	TBAB:EG 1:3	C(+)^d^/Pt(−)^c^	15 mA r.t., 2 h	Deoxygenative sulfonylation	[99]
15	ChCl:EG 1:5, 1:10, 1:20 + CH_3_CN	Pt(+)/Ni‐Co@C(−)^e^	80 mA r.t., 6 h	Lignin model reductive cleavage	[100]
16	ChCl:EG 1:2 +10% v/v H_2_O	Pt(+)/Cu(−)	−1.7 V versus Ag/AgCl r.t., 20 h	Lignin model oxidative cleavage	[101]
17	ChCl:MeU 1:2	Zn(+)/Cu(−)^f^	10 mA 50°C, 2.5 F	Lignin model reductive cleavage	[102]
18	ChCl:Gly 1:2	Pt(+)/Cu@C(−)^g^	−1.2 V versus SCE	Furfural reduction	[103]

a Graphite.

b Deposited on a screen‐printed graphite electrode.

c Deposited on a glassy carbon electrode.

d Carbon felt.

e Deposited on a Ni electrode.

f Reticular vitreous carbon.

g Deposited on a carbon plate electrode.

Although not strictly falling into the domain of organic electrosynthesis, it is worth mentioning that electrochemical conditions in DESs are gaining a relevant role also for the activation of small molecules, widely available on Earth but usually quite unreactive (such as CO_2_, N_2_, or O_2_), with applications in the fields of energy production and conversion, and of platform chemicals synthesis. Such transformations notably include, among others, water splitting, with concurrent HER and oxygen evolution reaction (OER) [134, 135], and CO_2_ reduction reaction (CO_2_RR) [66, 136, 137]. The latter encompasses also some organic reactions, which have been reported in the present section (see Schemes 7, 9–11, and 13). Recent comprehensive reviews have elegantly covered the topic of electrocatalysis in DESs for small molecules activation [65, 138], and some of the examples will be mentioned in the next sections of the present review, as applications of the materials obtained through inorganic synthesis in DESs.

## 4 Electrosynthesis of Metal Nanoparticles, Films, and Alloys

Among their electrochemical applications, DESs have found wide use as media for the preparation of metal NPs, alloys, films, and coatings by electrodeposition [64, 67, 120, 139]. The majority of reports concerning electrodeposition in DESs employs a quaternary ammonium halide (usually ChCl) with a HBD, i.e., an amide, an alcohol, or a carboxylic acid [70]. The methodology relies on the distinctive properties of DESs in terms of conductivity, the relatively wide potential windows, and high solubility of metal ions [120]. Most importantly, DESs play an important shape‐directing role in the nucleation and growth of NPs, defining their overall morphology and size distribution [64, 140, 141, 142, 143].

Another key advantage of using DESs concerns the possibility to control metal redox potentials in relation to the broad variety of formulations and the ability to handle water‐sensitive species, as well as guaranteeing the dissolution and stability of all the metal ion intermediates during the electrodeposition process [120]. Claims have also been made about the remarkable stability of NPs obtained with electrodeposition from DESs compared to other systems, which might be linked to their ability to effectively remove surface contaminants and to prevent the aggregation of the forming NPs [144]. A number of reviews have covered the developments in the preparation of nanomaterials in DESs, including electrochemical methods [64, 145, 146, 147, 148, 149]. Several reports on the electrodeposition of metal NPs from DESs are intended for studies of further electrocatalytic applications. In this section we first highlight a selection of recent advances reported in this latter field, and then showcase a few interesting cases of applications in other fields or fundamental studies on NPs growth and morphology control (Table 2).

### 4.1 Electrocatalytic Applications—Hydrogen Evolution, Oxygen Evolution, Conversion of Organic Compounds, and CO_2_

Electrodes of various materials have been modified with metal NPs of Ni, Fe, Co, Cu, Au, Pd, Ru, and have been tested for their electrocatalytic activity in water splitting reactions.

The synthesis of Ru NPs on cathodically treated stainless steel mesh (CSS) electrodes in ChCl:U 1:2 has been reported (Table 2, entry 1) [150]. An exhaustive electrochemical study was performed on the electrodeposited Ru NPs@CSS and revealed good stability and activity for HER in acidic medium. The use of a DES in this process represents a nontoxic alternative to cyanide‐based electrolytes that are commonly employed in the electrodeposition of noble metals. In addition, other general advantages related to the opportunity of operating in nonaqueous solutions consist in minimizing the formation of hydroxides as impurities, allowing for processes to occur at lower current intensity, and avoiding strong hydrogen evolution in the deposition phase.

The electrodeposition of Ni and Fe NPs on stainless steel mesh has been recently carried out in the same type of eutectic mixture (ChCl:U 1:2) [151]. Porous and spherical structures were observed and a large active surface was estimated for the resulting Ni–Fe@SS modified electrodes, which demonstrated significant activity when evaluated in HER, OER in alkaline medium (Table 2, entry 2).

Another contribution from the group of Sethuraman about the modification of stainless steel surfaces by metal electrodeposition in ChCl:U 1:2 concerned the preparation of Ni–Co alloy NPs (Table 2, entry 3) [152]. The morphology of the resulting Ni–Co@SS material was studied, revealing highly crystalline, spherical‐shaped NPs. An evaluation of the Ni–Co@SS electrode in HER and methanol oxidation (MOR) in alkaline media was carried out, demonstrating excellent catalytic activity and stability. Once more, the benefits of using a DES include avoiding hydrogen evolution during the formation of metal surfaces, compared to what is observed in aqueous phases, thus improving their final morphology.

Successive work involved the electrodeposition of a Ni–Cu alloy on stainless steel while investigating the effect of HBD variation in four different DES systems (Table 2, entry 4) [153]. The tested mixtures were prepared from ChCl and Ox, U, EG, and citric acid as HBD components, allowing for variation of both viscosity and hydrogen activity of the electrodeposition media. A uniform Ni–Cu coating was achieved with the use of ChCl:EG 2:1, while the deposition processes in other systems (DESs of higher viscosity) were most likely affected by reduced mass transport and ionic mobility. The variation in the obtained morphologies, attributed to the different HBDs in the DES, influenced the electrocatalytic performance of the synthesized materials in water splitting and MOR, with higher activity associated with the dendritic structure observed for Ni–Cu deposited in ChCl:EG.

**FIGURE 6:**
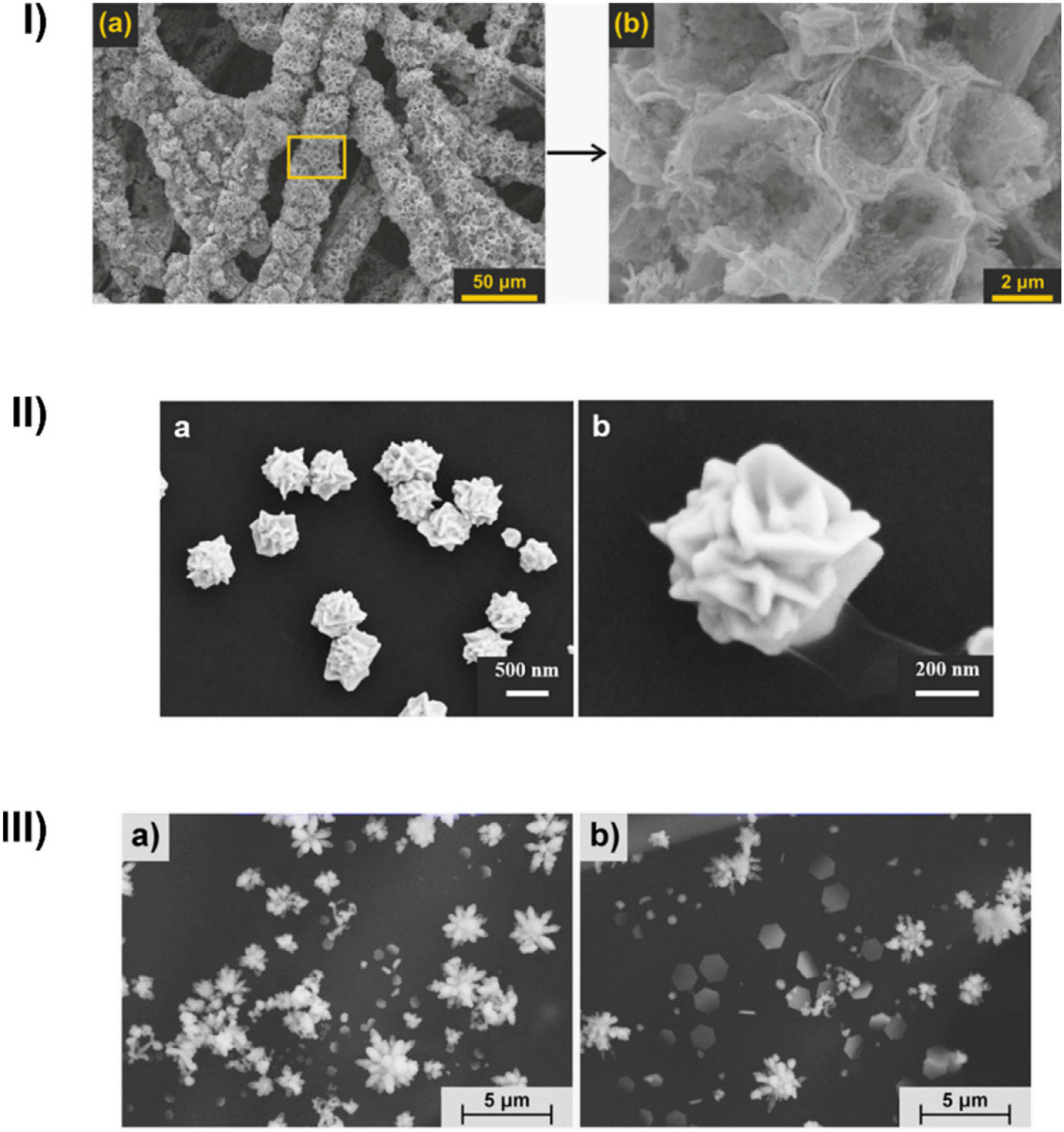
Examples of metal nanostructures obtained by electrodeposition from DES‐based electrolytes. SEM micrographs of: (I) Ni–Ru electrodeposited from ChCl:EG 1:2 containing H_2_SO_4_ and LiCl, reproduced from ref. [154] (2024, ACS, licensed under CC‐BY 4.0); (II) Au–Pt nanoflowers from ChCl:EG 1:2 + 10% H_2_O, reproduced from ref. [157] (2018, Springer Nature, licensed under CC‐BY 4.0); and (III) Cu clusters, and Cu_*x*_S hexagons from ChCl:EG 1:2 and 1:3, containing H_2_SO_4_, reproduced from ref. [166] with permission. 2025, Royal Society of Chemistry.

Still in the context of advancing clean energy technologies such as water electrolysis for hydrogen production, the synthesis of Ni–Ru materials with porous and branch‐like structures (Figure 6, I) has been accomplished by electrodeposition on carbon fiber paper in ChCl:EG 1:2 (Table 2, entry 5) [154]. Some of the ruthenium‐doped materials were transformed into Ni–Ru–Se by thermal selenization in order to enhance their electrochemical properties in HER. The advantages of the DES medium in this case are mostly associated with a wide electrodeposition potential window and low sensitivity to environmental conditions such as air and moisture.

In the same domain of developing electrocatalysts for hydrogen evolution, the electrodeposition of a film of nano‐Ni on Cu had been described in 2018, using both ChCl:U and ChCl:EG DESs [155]. The Ni coating nanostructures were found to be tuneable according to the use of different DESs and electrodeposition conditions, where the nano‐Ni@Cu prepared in ChCl:EG 1:2 displaying a needle‐like morphology demonstrated the highest activity in HER (Table 2, entry 6).

Furthermore, DESs obtained from metal chlorides and L‐serine were reported as a novel platform for the deposition of Ni, Fe, and Ni–Fe alloys on a nickel foam substrate, toward highly active OER electrocatalysts. The use of the metal salt/amino acid DES was defined as an environmentally friendly electrolyte alternative, associated with the potential to achieve high metal ion concentrations while enhancing deposition efficiency through the formation of coordination compounds (Table 2, entry 7) [156].

**SCHEME 21:**
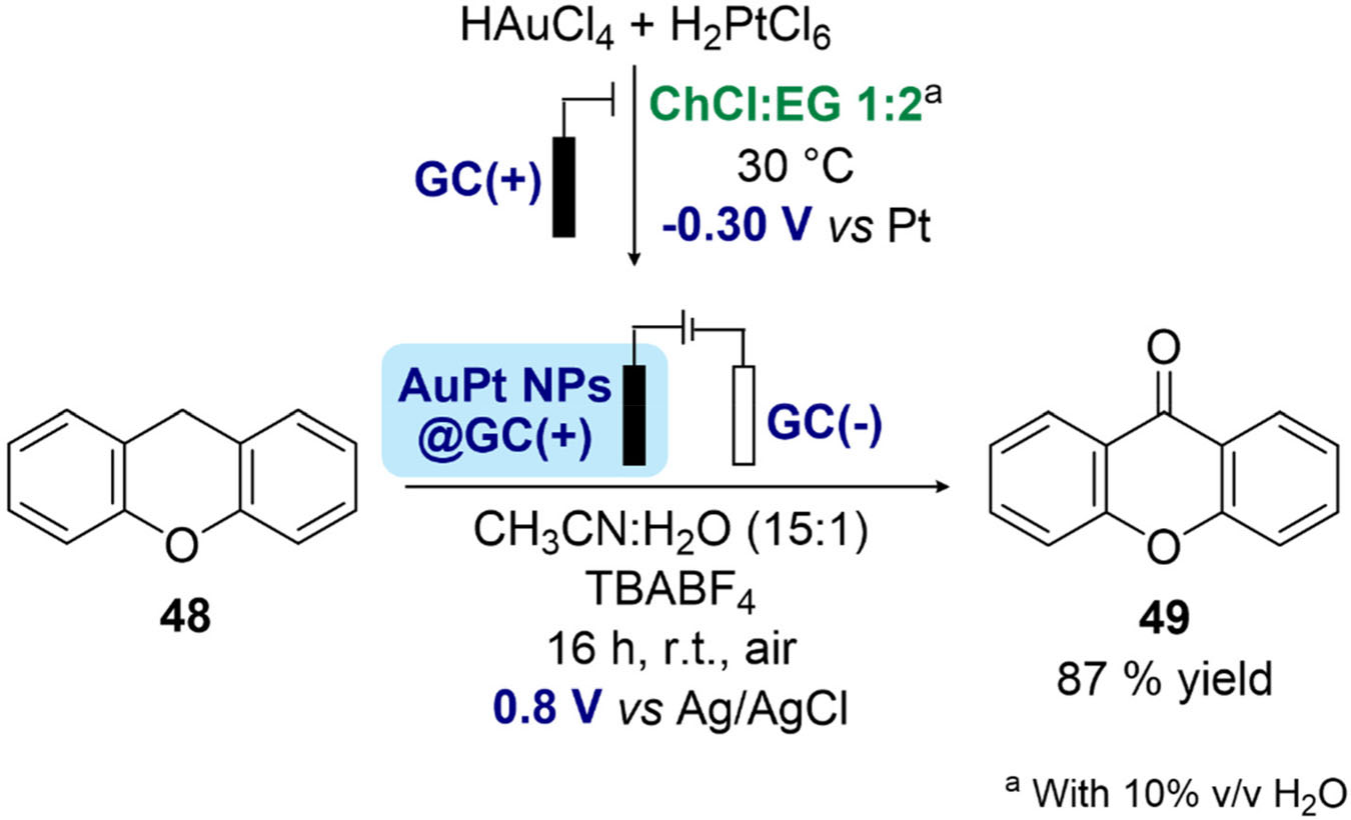
Electrosynthesis of AuPt NPs on glassy carbon electrode, and application in the electrosynthesis of xanthone from xanthene [157].

Interestingly, some examples also describe the activity of modified electrodes in the electro‐oxidation of organic compounds, such as xanthene (Table 2, entry 8) [157]. Indeed, NPs of a Au–Pt alloy with flower‐like structures (Figure 6, II) have been synthesized from HAuCl_4_ and H_2_PtCl_6_ in ChCl:EG 1:2 with a moderate addition of H_2_O (10% v/v, Scheme 21). The process occurred in an electroreductive step at low potential (−0.3 V vs. Pt *quasi*‐reference electrode) and low temperature (30°C), where the DES acts as a solvent and also defines the geometry of the NPs. Major effects on the material's morphology were observed while operating at higher water contents (50%–80%), thus disrupting the intrinsic characteristics of the DES environment, or when the applied potential was −0.7 V (vs. Pt *quasi*‐reference electrode), both causing the formation of irregular quasi‐spherical aggregates. Glassy carbon electrodes as‐modified with the Au–Pt flower‐like NPs were used as anode for the electro‐oxidation of xanthene **48** to xanthone **49** (Scheme 21) at a low constant potential (0.8 V vs. Ag/AgCl) and at room temperature, providing milder reaction conditions compared to traditional organic synthesis methods for this class of compounds of biological and pharmacological interest.

Other works have reported the electrosynthesis of Pd NPs using ChCl:U and ChCl:EG for the electrochemical oxidation of formic acid and different alcohols in the framework of the development of new fuel cells (Table 2, entries 9 and 10) [158, 159]. In view of preparing a catalyst for the electrochemical reduction of CO_2_ to formic acid, a Ga–Sn liquid alloy was electrodeposited on a copper foam substrate in ChCl:U 1:2 (Table 2, entry 11) [160]. When applied to CO_2_ conversion in a KHCO_3_ solution, the catalyst achieved a Faradaic efficiency of 90%. The DES was beneficial to the Ga and Sn electrodeposition process, both in suppressing HER and in tuning the metal ion concentrations to incorporate a high Ga content (91.6%) in the final alloy.

### 4.2 Energy Storage and Other Applications

The preparation of composites for energy storage applications by electrodeposition of Ag NPs on multiwalled carbon nanotubes (MWCNTs), carried out in ChCl:Gly 1:2, was reported by Anicai, Pereira et al. [161], with the DES acting as the electrolyte at room temperature and without requiring any previous modification of the CNTs surface (Table 2, entry 12). The use of the DES constitutes a cheaper, eco‐friendly alternative to conventional ILs, whilst aqueous systems display limitations of poor CNTs dispersion and electrochemical stability. The method was effective in controlling the NPs’ size, and the attachment of well‐defined Ag NPs to the CNTs walls resulted in a significant increase in capacitance for glassy carbon electrodes modified with such composites.

Processes of electrodeposition from DES for energy storage and conversion applications have been recently reviewed by Darband and coworkers, analysing in detail the effects of additives and water content towards nucleation processes, current efficiencies, and the resulting morphologies of deposited materials in relation to variations, e.g., in conductivity and viscosity of the electrolytes [172].

The minimization of hydroxides formation, the chance to operate at lower current intensities, and circumventing hydrogen evolution in the deposition are key advantages related to the use of DESs also in the case of Zn–Ni alloys deposition in ChCl:EG 1:2, achieving tuneable Ni content, for use in corrosion protection coatings (Table 2, entry 13) [162].

Besides examples of Ni deposition intended for HER, self‐supported Ni_3_S_2_ porous spheres have been synthesized on Ni foam for high‐performance supercapacitors [146]. First, Ni NPs were electrodeposited on the 3D support using ChCl:EG DES, in order to increase the active surface of the nanomaterial, then, the surface layer was converted into Ni_3_S_2_ by a sulfurization process, exhibiting a high specific capacitance and electrochemical stability (Table 2, entry 14) [163].

In a study by Gupta and coworkers, palladium NPs of controlled size, shape and dispersion have been prepared by electrodeposition on Au electrodes in ChCl:U 1:2, delivering a highly active and stable nanomaterial for the detection of ultra‐trace uranyl ions (down to 3.4 nM) in natural water samples (Table 2, entry 15) [164]. The Pd NPs, which exhibited an optimal electrocatalytic performance, could be obtained with a defined cauliflower‐shaped morphology by adjusting the water content in the DES to 5%. Furthermore, density functional theory calculations aided to gain insights into the great sensitivity for uranyl ion detection, an application that holds high relevance in environmental monitoring and nuclear safety.

The electrodeposition of hexagonal zinc nanostructures on TiO_2_ nanotube arrays (NTAs) was recently realized by Amani Hamedani and coworkers using ChCl:EG 1:2 containing ZnCl_2_ as the electrolyte (Table 2, entry 16) [165]. The obtained Zn NPs were found to improve the overall electrochemical properties of the TiO_2_ NTAs, which were proposed as promising electrocatalytic materials for implantable sensors.

DESs were also employed in the preparation of transition metal sulfides of interest as semiconductors. Specifically, recent work by Brzózka and Szczerba demonstrated the coelectrodeposition of Cu and S from ChCl:EG 1:2 producing various structures, including both S‐doped Cu clusters and Cu_*x*_S hexagons (of up to 23.8% S local content, Figure 6, III) when acidifying the electrolyte with H_2_SO_4_ (Table 2, entry 17) [166].

### 4.3 Fundamental Studies On Nucleation, Growth, and Morphology Control

A coelectrodeposition method in DES (ChCl:U 1:2) was employed to coat glassy carbon electrodes with bimetallic Cu and Au nanostructures (Table 2, entry 18) [167]. The materials were characterized and subjected to a Pb UPD (lead underpotential deposition) test, indicating a highly extended surface area, beneficial to their electrochemical activity. The effect of using a DES is linked to controlling the material morphology, with prospective applications in the preparation of multimetallic electrocatalysts.

In the field of the electrodeposition of reactive rare earth elements, recent work by the group of Palomar–Pardavé described a detailed study of the electrochemical nucleation and growth of neodymium onto a glassy carbon electrode from a Nd(III) complex precursor in ChCl:U 1:2 (Table 2, entry 19) [168]. Kinetic parameters for the electrodeposition process (such as nucleation frequency, active site density, and diffusion coefficients) were derived as functions of potential and temperature by means of current density transients analysis with physicochemical models accounting for multiple contributions. The nucleation models proposed in the DES system were validated by morphological characterization of the nanostructured Nd, which was found to contain Nd^0^ as well as oxides and hydroxides. Such insights expanding the knowledge about rare earth metal electrodeposition from DESs can moreover aid in defining optimal parameters for efficient electrochemical metal recovery processes.

The same group later investigated the mechanisms and kinetics of the electrosynthesis of Pd–Ag alloy NPs on glassy carbon from ChCl:EG 1:2, both for bare and polypyrrole‐modified electrodes (GC/PPy). The DES and PPy were indicated to act in synergy to control the morphology and uniformity of the fabricated nanostructures (Table 2, entry 20) [169].

A recent contribution by Hartley and Abbott describes the use of a CaCl_2_‐based DES (CaCl_2_ · 6H_2_O:EG 1:1) for the codeposition of Nd and Fe onto copper foil electrodes. It is worth to emphasize how the smaller size of the Ca^2+^ solvent cations, compared to the more common ChCl:EG 1:2 DES, might be responsible for the feasibility of electrodeposition of Fe^3+^ and Nd^3+^ species, due to a lower enrichment in bulky components at the electric double layer at cathodic potentials (Table 2, entry 21) [170].

Also, Zn nanorods of large sizes have been electrodeposited on porous alumina through an innovative two‐step process to sequentially promote the nucleation and growth of the structures using 0.5 M ZnCl_2_ in ChCl:EG (Table 2, entry 22) [171].

Moreover, the work of Doneux, Ustarroz, and coworkers is particularly relevant in this area, as they have extensively investigated nucleation and electrosynthesis mechanisms in DESs for metals such as Ag, Pd, Te, and alloys [70, 173, 174, 175, 176].

**TABLE 2:** Summary of the utilization of DESs in the electrosynthesis of metal NPs, films, and alloys and their applications.

Entry	**DES/DES‐like** **medium**	Electrodeposited metal/alloy	Substrate	Application	Ref.
1	ChCl:U 1:2	Ru NPs	SS	HER in acidic medium	[150]
2	ChCl:U 1:2	Ni–Fe NPs	SS	HER, OER, and water splitting in alkaline medium	[151]
3	ChCl:U 1:2	Ni–Co NPs	SS	HER and MOR in alkaline media	[152]
4	ChCl:EG 2:1	Ni–Cu alloy	SS	Water splitting and MOR	[153]
5	ChCl:EG 1:2	Ni–Ru	Carbon Paper	HER	[154]
6	ChCl:EG 1:2	nano‐Ni film	Cu	HER	[155]
7	NiCl_2_·6H_2_O, FeCl_3_·6H_2_O:L‐serine	Ni, Fe, and Ni–Fe	Ni Foam	OER	[156]
8	ChCl:EG 1:2 + 10% H_2_O	Au–Pt NPs	GC	Electrooxidation of xanthene	[157]
9	ChCl:U 1:2	Pd NPs	GC	Electrooxidation of formic acid	[158]
10	ChCl:EG 1:2	Pd NPs	GC	Electrooxidation of formic acid	[159]
11	ChCl:U 1:2	Ga–Sn liquid alloy	Cu Foam	CO_2_ reduction to HCOOH	[160]
12	ChCl:Gly 1:2	Ag NPs	MWCNTs	Composites for energy storage	[161]
13	ChCl:EG 2:1	Zn–Ni alloy	Steel	Corrosion protection coatings	[162]
14	ChCl:EG 1:2	Ni_3_S_2_ porous spheres	Ni Foam	High‐performance supercapacitors	[163]
15	ChCl:U 1:2 + 5% H_2_O	Pd NPs	Au	Detection of ultra‐trace uranyl ions	[164]
16	ChCl:EG 1:2	Hexagonal Zn NPs	TiO_2_ NTAs	Proposed for implantable sensors	[165]
17	ChCl:EG 1:2 + H_2_SO_4_	Cu and S (S‐doped Cu clusters, Cu_*x*_S hexagons)	Carbon Paper	Semiconductors, optoelectronic devices	[166]
18	ChCl:U 1:2	Cu–Au NPs	GC	—	[167]
19	ChCl:U 1:2	Nd	GC	—	[168]
20	ChCl:EG 1:2	Pd–Ag NPs	GC and GC/PPy	Several proposed, e.g., ethanol oxidation, hydrogen storage/production, etc.	[169]
21	CaCl_2_ · H_2_O:EG, 1:1	Fe, Nd and Fe–Nd	Cu foil	—	[170]
22	ChCl:EG 1:2	Zn nanorods	Porous alumina	—	[171]

## 5 Electrosynthesis of Metal Oxides

DESs have primarily been used in the dissolution of metal oxides, rather than their synthesis, particularly in the context of extraction and processing of metals from both naturally occurring sources and waste materials such as spent batteries [59, 60]. However, a number of reports concerning the electrochemical synthesis of metal oxides using a DES‐based electrolyte have appeared in the literature, and we herein describe some of these examples. In addition to the preparation of metal oxides, it is worth mentioning that a one‐step electrochemical approach in a mixture of ChCl:U 1:2 and water, comprising both oxidation and exfoliation processes, has also been reported for the synthesis of hydroxylated boron nitride nanosheets (OH‐BNNSs) to be employed in solid‐state flexible supercapacitors [177, 178].

Nanostructured NiO thin films were obtained by electrodeposition on ITO glass using ChCl:EG 2:1 as electrolyte in the presence of a Ni salt precursor and oxidizing agents (Table 3, entry 1) [179]. A compact film with small particle size (nanogranules, 2–6 nm) was prepared when operating at 90°C, due to the viscosity change of the DES, which resulted in improved electrochromic performance of the material compared to those obtained at lower temperature, with potential applications in optoelectronic devices. This type of one‐step fabrication procedure in DES grants the circumvention of the oxidative annealing of deposited Ni films. The electrochemical preparation of cobalt oxidized species over glassy carbon and nickel substrates in ChCl:Gly 1:2 was also reported [180]. In the presence of a controlled amount of water, the method produced a composite material with a polyglycerol porous structure integrating the metallic oxides, which could be exploited as multifunctional heterogeneous catalyst for OER in alkaline solutions or as nonenzymatic glucose sensor (Table 3, entry 2) [181]. This approach involves an initial deposition of metallic Co from the solution, which then promotes the formation of cobalt oxide and hydroxide species via reduction of water present as supporting HBD in the DES, preventing the need for an additional thermal treatment.

**TABLE 3:** Summary of the utilization of DESs in the electrosynthesis of metal oxides and their applications.

Entry	**DES/DES‐like** **medium**	Electrodeposited metal oxide	Substrate	Electrocatalytic application	Ref.
1	ChCl:EG 1:2	NiO	ITO	Optoelectronic devices	[179]
2	ChCl:Gly 1:2	Co oxy/hydroxides	GC and Ni	OER in alkaline medium/Nonenzymatic glucose sensors	[180]
3	ChCl:U 1:2 ChCl:EG 1:2 ChCl:malonic acid 1:1	TiO_2_	Ti	—	[181]
4	ChCl:ZnCl_2_ 1:2 (as additive, 5%)	SnO	Sn	Wastewater treatment	[182]
5	ChCl:ZnCl_2_ 1:2 (as additive, 5%)	ZnO	Zn	Wastewater treatment	[183]
6	ChCl:U:EG	SnO_2_‐Sb	Ti/TiN	Wastewater treatment	[184]

Various ChCl‐based DESs were employed as electrolytes for the anodization of Ti in the presence of ammonium fluoride and water to prepare ordered TiO_2_ nanotubes (Table 3, entry 3) [185]. The effect of using diverse HBDs (U, EG, or malonic acid) was investigated, and the formation of NTs with different morphologies was observed depending on the electrolyte viscosity.

The anodization of Sn using ChCl:ZnCl_2_ 1:2 as the additive (5% v/v) in a plant extract derived from spent coffee grounds as electrolyte has also been reported (Table 3, entry 4) [182]. The electrosynthesis was aimed at the preparation of a SnO catalyst for the photodegradation of 2,4‐dichlorophenol in the context of wastewater treatment. The plant extract‐DES combination generated an oxide with excellent photodegradation efficiency and high superficial area, and its organized structure was attributed to a templating effect promoted by the DES component. The approach was highlighted as an environmentally friendly alternative of tin oxide fabrication, by both eliminating the drawbacks of using toxic aprotic organic solvents as *N*,*N*‐dimethylformamide and tetrahydrofuran, and by repurposing waste material. In a related work, the strategy was extended to the preparation of ZnO in the presence of ChCl:ZnCl_2_ as electrolyte to obtain a high‐performing catalyst for the photodegradation of chlorophenol derivatives in water remediation (Table 3, entry 5) [183]. Similarly, the ZnO sample of more uniform and well‐defined structure was achieved when using DES (5% v/v) in the electrolyte, compared to the bare plant extract, so that the DES was defined a structure‐directing agent.

In a recent work by Tang and coworkers, titanium‐based antimony‐doped tin dioxide anodes (Ti/TiN/SnO_2_‐Sb) were prepared by electrodeposition in a ternary DES of ChCl:U:EG followed by pyrolysis, as a strategy to improve their stability and obtain larger specific surface area (Table 3, entry 6) [184]. The electrodes displayed high activity and were applied to the electro‐oxidative degradation of hydroxychloroquine for wastewater treatment, reaching a removal rate of 85.5%.

## 6 Summary and Outlook

DESs have been proven as effective and versatile media for the electrosynthesis of metal NPs, alloys, and inorganic oxides. Their use enables precise control over the materials’ morphology, size, and chemical composition, in addition to replacing toxic or hazardous electrolyte components used in traditional methods. The ability to finely adjust the properties of DESs by varying their components can impact electrodeposition processes both in terms of the efficiency of metal recovery and the performance of the resulting materials. Electrodeposited materials synthesized in DESs demonstrated an enhanced electrocatalytic activity and stability for applications such as HER, OER, water splitting, organic electrosynthesis, energy storage, and environmental monitoring. Therefore, DESs have emerged overall as a powerful and sustainable platform for the controlled electrosynthesis of advanced functional nanomaterials for catalysis, energy, environmental, and sensing applications.

On the other hand, the electrosynthesis of organic compounds in DESs, although appearing to be a more recent and less mature field, demonstrates a very promising potential for the efficient and sustainable construction of complex molecular architectures, relevant to pharmaceutical and industrial applications, and for the conversion of biomass and renewable feedstock. The combination of the activating capabilities of DESs towards organic substrates, mainly through hydrogen bonding and Lewis acidity and basicity, with the unique electrocatalytic induction provided by current‐ and potential‐driven processes results in a positive synergistic effect on the reactivity, resulting in higher reaction rates and yields, at milder conditions. Remarkably, in most cases this dual activation makes it possible to obtain the desired products in the absence of any additional reagents, catalysts, and supporting electrolytes, improving the sustainability under the points of view of waste production and management, and costs. These parameters can be complemented by the recycling of the solvent and/or of surface‐modified electrodes; however, since the recycling procedure may introduce additional amounts of organic solvents, it is important to evaluate, on a case‐by‐case basis, whether its implementation actually provides a quantitative improvement, e.g., in terms of *green* metrics and feasibility. Furthermore, from the examples herein reported emerges the pivotal role of *ad hoc* solvent design, taking advantage of the broad tunability of DESs’ components, as a powerful tool enabling specific reactivity to efficiently take place, and as a central aspect for future development of DES‐based electrosynthetic methodologies.

Indeed, in terms of potential applications of electrosynthesis in DES, many classes of reaction have not been covered yet, and could represent possible room for sustainable innovation. In organic chemistry, the exploration of synthetic opportunities for functional and pharmaceutical compounds appears to be just at its beginnings, and in particular it could move towards the direction of constructing specialized carbon‐heteroatom bonds, such as carbon–phosphorus and carbon–silicon, which require a controlled electrical potential, and carbon–fluorine, which remains a privileged motif for several applications, despite perfluorinated compounds being gradually phased out due to toxicity concerns. At the edge between organic and inorganic chemistry, the synthesis of two classes of materials has yet not been investigated through electrosynthesis in DESs. The first one includes carbon‐based nanomaterials, such as porous carbons, graphene, and carbon nanotubes, which have a wide range of applications, particularly in the field of electrochemical energy storage (batteries and supercapacitors), but also in catalysis and biocatalysis. The second one is represented by MOFs and COFs, whose synthesis could be performed by direct growth on the electrode surface, in an electroinduced process.

Furthermore, our analysis of the currently available literature underlines that several mechanistic proposals are still based on a significant degree of speculation, lacking a detailed comprehension of the actual role of DES components, and DESs as a partially organised supramolecular entity, on the course of the reaction, and in some cases even lacking sufficiently grounded insights into the main events of electrochemical activation. In our opinion, future work on organic electrosynthetic methodologies would benefit from thorough electrochemical characterization, through cyclic voltammetry, of the blank solvents and of the behaviour of the substrate(s), including a comparison with the state‐of‐the‐art conventional solvents used for the reaction in question.

Overall, the use of DESs as media represents a highly valuable opportunity to improve electrosynthesis under both points of view of reactivity and sustainability. However, it ought to be emphasized how some common claims about their general properties might benefit from more detailed clarification, such as their effective low toxicity and sustainability profiles, particularly for newly proposed HBA:HBD mixtures, or how experimental parameters truly affect the width of their electrochemical window.

## Author Contributions

**Cristiana Margarita**: investigation, writing – original‐draft, visualization, data‐curation, writing – review‐editing. **Stefano Nejrotti**: data‐curation, visualization, writing – review‐editing, writing – original‐draft, investigation. **Alejandro** **Leal‐Duaso**: writing – review‐editing, validation, data curation. **Marta Feroci**: writing – review‐editing, funding‐acquisition, supervision, conceptualization. **Alessandra Sanson**: funding‐acquisition, writing – review‐editing, supervision. **Claudia Barolo**: funding‐acquisition, writing – review‐editing, supervision. **Matteo Bonomo**: conceptualization, funding‐acquisition, writing – review‐editing, project‐administration, supervision.

## Funding

This study was supported by Ministero dell'Università e della Ricerca (grant B53C24005960006, B53C22004060006, D13C22003520001) Agencia Estatal de Investigación (grant PID2021‐125762NB‐I00), Gobierno de Aragón (grant E37_23R).

## Conflicts of Interest

The authors declare no conflicts of interest.
